# Natural Histidine Derivatives—From Basic Research to Potential Applications in Cosmetics and Nutricosmetics

**DOI:** 10.3390/antiox15091118

**Published:** 2026-09-04

**Authors:** Edyta Gołaś, Mateusz Maciejczyk

**Affiliations:** 1 Students’ Scientific Club “Biochemistry of Lifestyle Diseases” at the Department of Hygiene, Epidemiology and Environmental Health, Medical University of Bialystok, 2c Mickiewicza Street, 15-233 Bialystok, Poland; edyta.golas@sd.umb.edu.pl; 2 Department of Hygiene, Epidemiology and Environmental Health, Medical University of Bialystok, 2c Mickiewicza Street, 15-233 Bialystok, Poland

**Keywords:** aging, oxidative stress, longevity, skin, ergothioneine, selenoneine, ovothiol A

## Abstract

Skin aging is a complex process influenced by oxidative stress, protein glycation, chronic inflammation, and increased extracellular matrix remodeling. Intensive research is underway on new anti-aging substances with multi-target mechanisms of action while maintaining safety and efficacy. Ergothioneine, selenoneine, and ovothiol A are natural histidine derivatives of marine origin, in which the oxygen atom of the hydroxyl group has been replaced with sulphur or selenium. In recent years, a broad spectrum of their biological activity has been demonstrated. Despite the well-documented antioxidant potential of these compounds, their anti-aging effects, particularly in terms of antiglycation and anti-inflammatory activity, remain insufficiently understood. This study presents the current state of knowledge regarding the biological activity of ergothioneine, selenoneine, and ovothiol A, and discusses available cosmetic preparations and dietary supplements containing these compounds. Meanwhile, significant research gaps have been identified regarding their potential use in the prevention and treatment of skin aging.

## 1. Introduction

Aging is a complex physiological process that encompasses a number of biochemical changes occurring in living organisms over time [1]. Aging leads to a gradual decline in the body’s performance, both in terms of physical and cognitive functions, and an increased susceptibility to disease [2]. This condition is a consequence of increasing cellular damage, a decline in tissue regenerative capacity, and, consequently, a progressive loss of organ function [3]. The most commonly used indicator of human age is chronological age, defined as the number of years since birth [4]. Although it is an objective and precise measure, it does not reflect the individual rate of aging. It is increasingly emphasized that chronological age does not always correlate with the actual health or functionality of the body [5]. Biological age refers to the degree of damage to cells, tissues, and organs, providing a more precise indicator of the advancement of the aging process [6,7]. Biological age, unlike age expressed in years, is dynamic and dependent on many factors. Genetic factors, lifestyle, exposure to environmental pollutants [8,9], and the presence of chronic diseases such as diabetes, cancer, or Hutchinson–Gilford Progeria Syndrome constitute important elements. They can accelerate the aging process [10,11]. This means that people who lead a healthy lifestyle and are free of chronic diseases may have a biological age significantly lower than their chronological age [9]. In the biology of aging, two mechanisms related to this process are distinguished: intrinsic (endogenous) aging and extrinsic (exogenous) aging. Intrinsic aging is related to the effects of time, genetic factors, and metabolic processes. Extrinsic aging, in turn, is associated with chronic exposure to harmful environmental factors. Both types of aging—endogenous and exogenous—overlap, leading to cumulative tissue damage, reduced regenerative capacity, and deterioration of individual organ systems [12]. The integumentary system, comprising the skin and its appendages [13], serves a crucial function as a protective barrier, separating the external environment from the body’s interior. Skin, as the only tissue, is in constant contact with various damaging factors, such as air pollution, viruses, bacteria, toxins, and ultraviolet (UV) radiation, and is therefore particularly susceptible to their harmful effects [14,15]. Clinically, skin aging manifests itself as wrinkles, furrows and discoloration and loss of skin elasticity. Moreover, the age-related prolongation of epidermal turnover time contributes to slower epidermal renewal and desquamation, compromised barrier homeostasis, and a reduced capacity for timely repair and regeneration following injury [16]. It is also worth noting that the age-related decline in collagen production contributes to progressive loss of dermal thickness, elasticity, and mechanical strength, thereby promoting skin laxity and wrinkle formation [17]. These features are considered aesthetic defects, albeit they indicate a deterioration in skin physiology and function. Skin aging is underpinned by increased production of reactive oxygen species (ROS) and nitrogen species (RNS) and the associated oxidative and nitrosative stress, non-enzymatic attachment of reducing sugars to proteins (protein glycation), increased production of pro-inflammatory cytokines and chemokines (inflammation), and impaired remodeling of the extracellular matrix [18,19,20,21,22,23,24]. Hence, contemporary anti-aging strategies are based primarily on antioxidant activity, photoprotection, hydration, stimulation of collagen synthesis, modulation of cellular processes, modern regenerative treatments, and a healthy lifestyle that includes aspects such as diet, sleep, physical activity, stress reduction, and supplementation [25,26,27,28]. The best-known and most frequently used substances in cosmetics and anti-aging treatments include vitamin C [29], vitamin E [30], coenzyme Q10 [31], retinoids [32] and polyphenols such as resveratrol, quercetin [33] or ferulic acid [34]. Despite the availability of numerous compounds with well-known anti-aging properties, the search is ongoing for new compounds that exhibit greater efficacy and target key mechanisms of skin aging.

In this review, we will take a closer look at the skin aging process and the possibilities of slowing it down using natural bioactive compounds. Thiols constitute a large group of natural substances used in cosmetology. These are organic chemical compounds (alcohol derivatives) in which the oxygen atom of the hydroxyl group has been replaced with sulphur. These compounds are believed to have antioxidant, anti-inflammatory, and detoxifying effects, which are due to the presence of a free sulfhydryl group (-SH) [35]. However, little is known about the anti-aging properties of histidine derivatives containing sulphur. Natural histidine compounds of marine origin include ergothioneine (containing sulphur at the C2 position of the imidazole ring of histidine) [36,37,38], the recently discovered ergothioneine analogue, selenoneine, which has (instead of sulphur) a selenium atom at the C2 position of the imidazole [39], and ovothiol A (containing sulphur at the C5 position of the imidazole) [40,41,42]. The aim of this study is to characterize ergothioneine, selenoneine, and ovothiol A and assess their potential role as anti-aging ingredients. Analysis of the properties of these compounds may contribute to the development of new cosmetic strategies aimed at molecularly protecting the skin against the effects of premature aging.

## 2. Histidine-Derived Alkaloids

**Figure 1:**
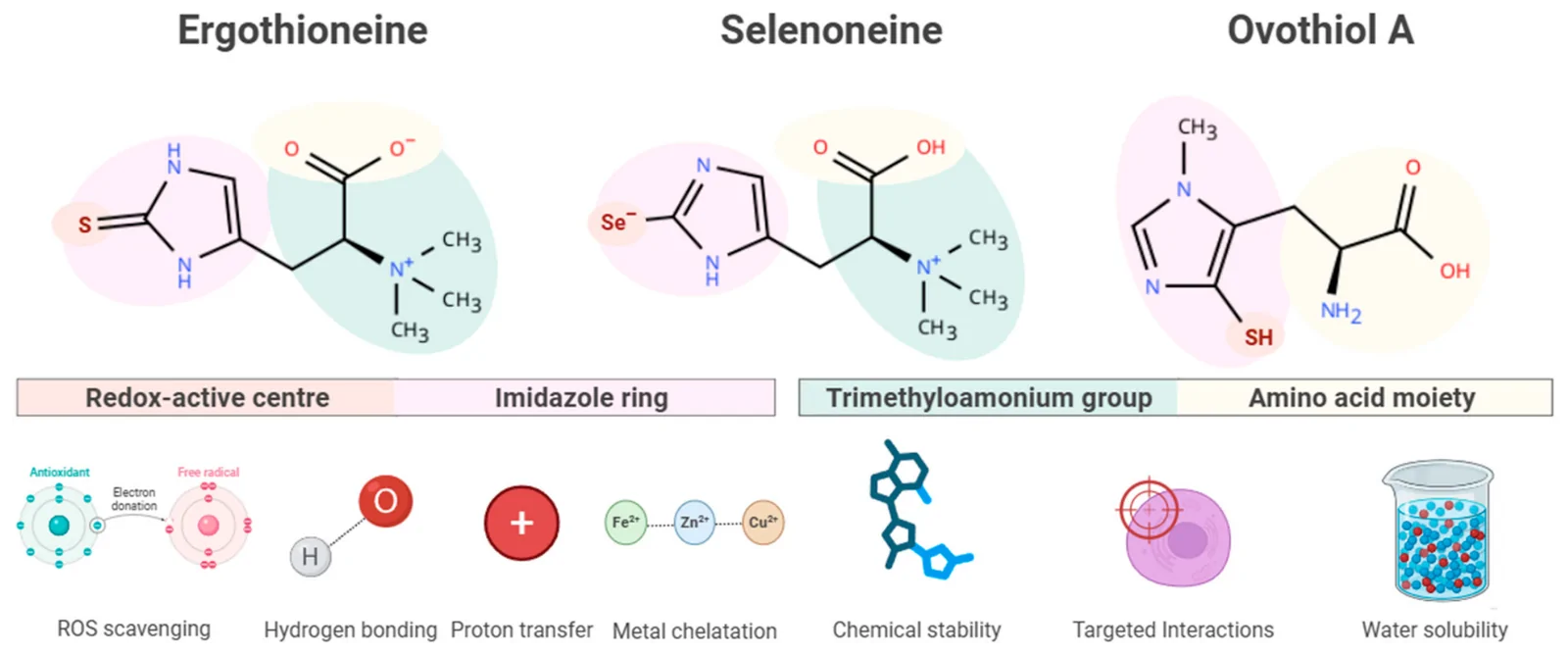
Biological properties of ergothioneine, selenoneine, and ovothiol A in relation to their chemical structures. Created in BioRender. Maciejczyk, M. (2026) https://BioRender.com/mdjjfdg.

Histidine-derived alkaloids are rarely encountered in nature, though they have attracted the attention of researchers due to their broad spectrum of biological activity [43,44] (Figure 1). Histidine derivatives containing an imidazole ring in their structure are of particular interest [45]. Among them, derivatives containing sulphur or selenium are distinguished [46,47,48]. Selenium replaces sulphur in two well-known amino acids: selenocysteine and selenomethionine. Although selenium is highly similar to sulphur in terms of its atomic structure and participation in redox reactions, sulphur as a macroelement is common in nature, while the occurrence of selenium in the natural environment is rare [48,49,50,51,52,53].

### 2.1. Ergothioneine

Ergothioneine (2-thiol-L-histidine betaine) [36] is a natural histidine derivative containing a thiourea moiety (C=S) resulting from the substitution of a sulphur atom at the C2 position of the imidazole ring [36,37]. Due to the presence of the -SH group, ergothioneine is classified as a thiol compound. In aqueous solutions, ergothioneine exists as a tautomer between the thione and thiol forms. At physiological pH, the sulphur atom donates a proton (H+), which changes the structure of ergothioneine from a thiol to a thiourea [54]. The predominance of the thione form over the thiol form at physiological pH makes ergothioneine highly resistant to autoxidation in aqueous solutions, distinguishing it from typical thiols such as glutathione [36,55]. Ergothioneine has a low molecular weight and contains several polar functional groups. The presence of a carboxyl group and a trimethylammonium cation gives it a betaine character, ensuring its high water solubility, chemical stability, and the ability to interact specifically in biological environments [56,57]. Furthermore, the presence of sulphur and nitrogen atoms in its structure allows ergothioneine to interact with metal ions and reactive oxygen species [58]. This type of chemical structure is not often found in natural compounds, especially with the coexistence of the thiol and betaine forms. The unusual chemical structure of ergothioneine provides a wide range of biological activities. The unusual chemical structure of ergothioneine provides a wide range of biological activities including ROS scavenging, hydrogen bonding, proton transfer, metal chelation, chemical stability, targeted interactions, and water solubility [56,58,59].

#### 2.1.1. Ergothioneine Biosynthesis

**Figure 2:**
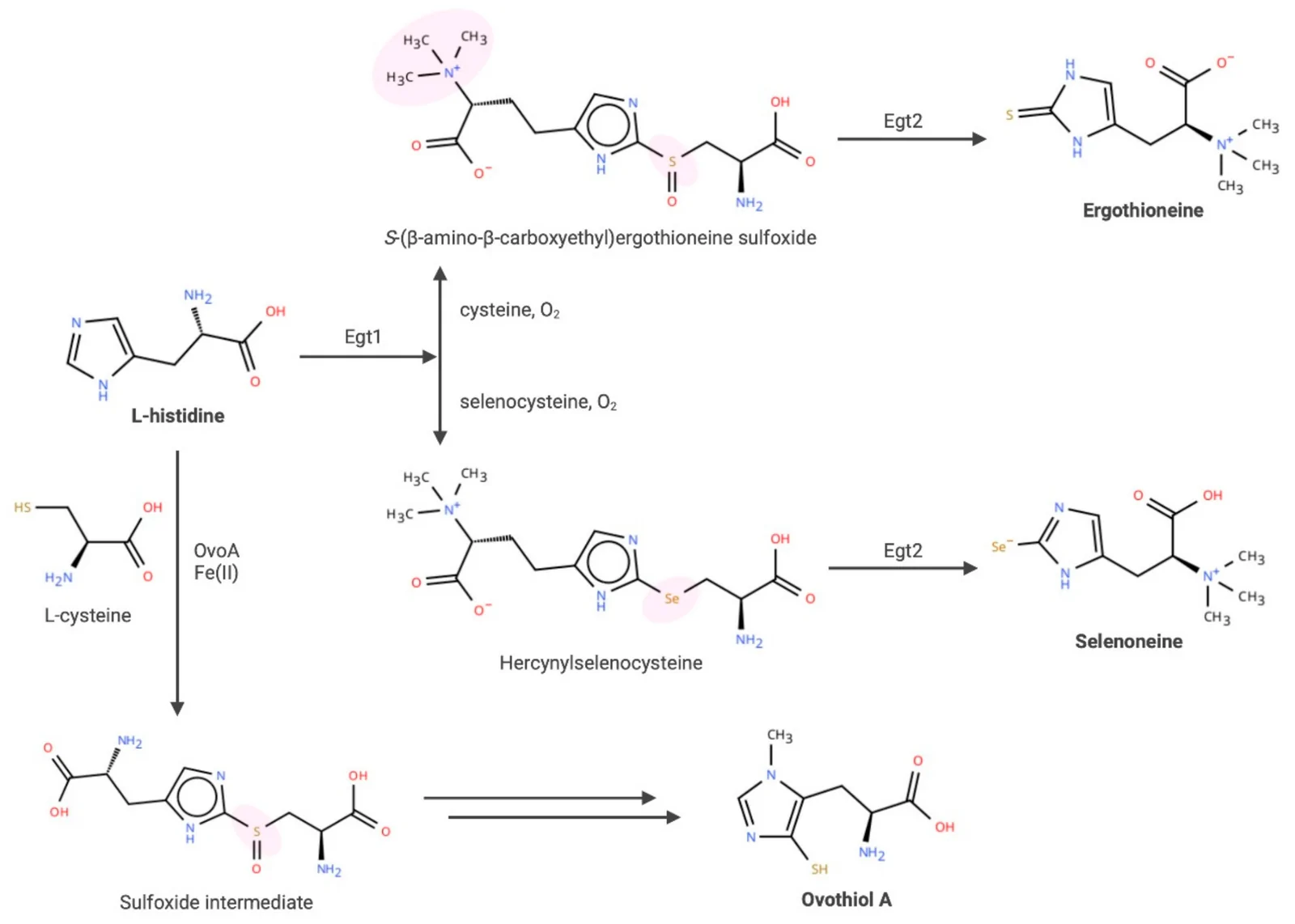
Simplified biosynthetic pathway of ergothioneine, selenoneine, and ovothiol. Created in BioRender. Maciejczyk, M. (2026) https://BioRender.com/q9is7a2.

The biosynthesis of low-molecular-weight thiol compounds is widespread in prokaryotic and eukaryotic organisms [60]. Ergothioneine is the best-studied sulphur analog of histidine, albeit its biosynthetic pathways have only been discovered in recent decades [61]. So far, ergothioneine biosynthesis is known to occur exclusively in bacteria, cyanobacteria, and fungi. Nevertheless, it should be noted that small amounts of ergothioneine have been detected in plants and animals, including significant physiological amounts in humans [62]. A molecular pathway for ergothioneine biosynthesis in animals and humans does not exist [63]. Ergothioneine is obtained from food, and its transport is facilitated by the membrane human novel organic cation transporter 1 (OCTN1), encoded by the *SLC22A4* gene. Transporter OCTN1, is widely distributed in many cells and tissues [64]. Despite extensive research, there is a significant gap in knowledge about ergothioneine biosynthesis in plants. Currently, the most plausible theory is that plants obtain ergothioneine from soil bacteria and fungi via their roots as part of a mycorrhizal symbiosis [54]. Ergothioneine has been detected in the mycoheterotrophic plant *Gastrodia elata*, whose life cycle depends on the presence of symbiotic fungi [65]. There are several distinct pathways for ergothioneine biosynthesis: oxygen-dependent and oxygen-independent. The most common mechanism of biological ergothioneine synthesis involves oxygen-dependent C-S bond formation, catalyzed by iron-dependent sulfoxide synthases (EgtB and Egt1) [61,66] (Figure 2) The EgtB-dependent mechanism can be illustrated by the example of actinomycetes, in which ergothioneine biosynthesis involves five steps [67]. This process begins with the N-α-trimethylation of histidine to hercynin, catalyzed by S-adenosylmethionine (SAM) EgtD [68]. Simultaneously, EgtA or glutamylcysteine ligase condenses glutamate and cysteine, producing γ-glutamylcysteine as a source of the thiol group [69].

In the next reaction, catalyzed by sulfoxide synthase EgtB, hercynin and γ-glutamylcysteine are conjugated in an oxygen-dependent manner, resulting in the formation of sulfoxide 1 [70]. Next, the glutamyl group is removed from sulfoxide 1 by amidotransamidase EgtC, resulting in sulfoxide 2 [70]. In the final step, pyridoxal 5′-phosphate (PLP) β-lyase EgtE catalyzes the cleavage of the C-S bond in sulfoxide 1, releasing ergothioneine as the final product [70]. The mechanism of ergothioneine biosynthesis, dependent on the bifunctional enzyme Egt1, has been observed in *S. pombe* yeast cells [71]. As in the case of actinomycetes, the starting substrate is L-histidine. In the first step, Egt1 catalyzes the trimethylation of the amino group of histidine to form hercynin using SAM as a methyl donor. The same enzyme then catalyzes the conjugation of hercynin with L-cysteine in the presence of molecular oxygen, resulting in the formation of hercynylcysteine sulfoxide—a characteristic intermediate of the specific ergothioneine biosynthetic pathway [72,73]. Termination of ergothioneine biosynthesis is catalyzed by the enzyme Egt2, through cleavage of the C-S bond and elimination of the cysteine amino acid residue, leading to ergothioneine as the final product [66,74]. Since fungi lack the ability to produce the enzyme involved in the removal of the glutamyl fragment of the sulfoxide intermediate 1 found in the ergothioneine biosynthetic pathway in bacteria, they use cysteine, not γ-glutamylcysteine, as a thiol donor [66,74].

#### 2.1.2. Oral Bioavailability of Ergothioneine

Even though animals are unable to biosynthesize ergothioneine, it has been shown that this compound is efficiently absorbed and actively transported to many cells and tissues [64,75,76,77]. Since ergothioneine is a hydrophilic compound, it cannot cross cell membranes on its own, and therefore a specific transmembrane transporter protein is required for its entry into cells. Literature reports indicate that the OCTN1 is capable of transporting ergothioneine. For this reason, OCTN1 is referred to as the ergothioneine transporter [62]. The transport of low-molecular-weight compounds, carried out by OCTN1, plays a key role in their bioavailability and function in the body, which is why it is widely distributed in many cells and tissues [64]. *SLC22A4* gene knockout studies [78,79] have demonstrated the complete dependence of ergothioneine on OCTN1 [64,77,78,80,81]. Loss of OCTN1 in model organisms was not associated with death but increased susceptibility to oxidative stress and inflammation [78,79], which is consistent with the antioxidant and cytoprotective functions attributed to ergothioneine. Interestingly, a positive correlation has been demonstrated between ergothioneine concentration in the blood and average lifespan, which is why it has been called the longevity vitamin [82]. Ergothioneine from food is absorbed in the small intestine and distributed to cells and tissues via blood vessels [67,77,83]. Ergothioneine is characterized by high systemic accumulation and very low excretion. Cheah et al. demonstrated that after oral administration of ergothioneine to humans, it is rapidly absorbed and retained in the body, with a significant increase in its concentration in plasma and whole blood, while urinary excretion accounted for <4% of the administered ergothioneine [84]. The high uptake and high retention of ergothioneine suggest an important physiological function in the body. It has been shown that the higher the expression of OCTN1, the greater the amount of this compound accumulated [77]. OCTN1 is found in many tissues, including skin, liver, kidney, small intestine, bone marrow, stomach, brain, hematopoietic cells such as nucleated erythrocyte precursors, neutrophils and monocytes [85]. The ergothioneine transporter is also found in neurons, dendrites, and microglial cells of the brain [86]. Consequently, the ubiquitous expression of OCTN1 guarantees the wide distribution of ergothioneine in various tissues and organs. Ergothioneine content varies between tissues not only due to different OCTN1 expression but also due to the susceptibility of tissues to damage [78,87]. Ergothioneine remains at high levels, particularly in tissues and organs susceptible to oxidative stress and inflammation [78,87]. Under physiological conditions, the highest ergothioneine concentrations occur in the liver and blood. However, after oral administration, ergothioneine has been observed to accumulate most abundantly in the eyes, kidneys, spleen, intestines, bone marrow, and brain [81,88]. Literature data indicate no significant relationship between ergothioneine concentration and gender [89,90,91]. However, a decrease in ergothioneine concentration in whole blood and plasma with age has been observed [72]. The decrease in ergothioneine content with age may be related to changes in dietary habits as well as changes in *SLC22A4* gene expression [92,93]. Ergothioneine levels have been shown to be significantly reduced in patients with mild cognitive impairment [78,79,89,94], while no reductions in this compound have been observed in healthy individuals. These findings strongly suggest that reduced ergothioneine levels may be associated with neuropathy and progressive aging [78,79,89].

#### 2.1.3. Anti-Aging Properties of Ergothioneine

Oxidative stress is a key factor in skin aging; it damages cellular structures and exacerbates changes through glycation and inflammation [21,95]. A regular supply of an exogenous antioxidant compound, especially one that is absorbed in the small intestine or is well absorbed through the skin, could have positive anti-aging effects on the skin. Ergothioneine is a remarkable reducing agent; the reduction potential for its thiol group is +0.06 V [55,96,97] and is much higher than for glutathione (−0.25 V) [98]. Ergothioneine can detoxify and inhibit the formation of various ROS, including hydroxyl radicals [99,100,101], singlet oxygen [102,103,104,105,106,107], ozone [108], superoxide anions [109,110,111,112], peroxides [97,113], and hypochlorite [101,114]. Notably, Asmus et al. reported that in the presence of vitamin C, the oxidized form of ergothioneine is reduced back to its active state by vitamin C, leading to the formation of ascorbyl radicals [100]. This interaction between ergothioneine and vitamin C is similar to the previously observed cooperative interaction between vitamin E and vitamin C, which may contribute to significant biological redox protection [115]. Furthermore, ergothioneine has been shown to be a more potent free radical scavenger than classic antioxidants such as glutathione, uric acid, and trolox [116]. Remarkably, ergothioneine reacts with singlet oxygen substantially faster than vitamin C and L-histidine [117]. Ergothioneine provides strong antioxidant activity even at very low (nanomolar) concentrations, which constitutes its substantial advantage [118]. The presented conclusions point to ergothioneine as a highly important antioxidant. Nitrosative stress is also considered to play a significant role in skin aging. Nitrosative stress, through the action of reactive nitrogen species (RNS), further exacerbates damage to cellular structures and accelerates degradative processes in the skin [119,120]. Jung-Hee Jang et al. demonstrated that ergothioneine can attenuate β-amyloid-induced apoptosis by preferentially eliminating peroxynitrite in rat pheochromocytoma (PC12) cells in vitro [109]. Furthermore, the data presented by Alhalwani et al. clearly indicate the protective effect of ergothioneine, as it effectively inhibited the chemical modification of lactoferrin and lamb corneal lysate by reducing the formation of 3-nitrotyrosine after exposure to nitrating reagents such as peroxynitrite and tetranitromethane in an in vitro model [121]. A factor that exacerbates oxidative-nitrosative stress is the presence of transition metal ions, such as iron and copper, which can catalyze the formation of reactive oxygen and nitrogen species [122,123,124]. In this context, metal chelation is particularly important—limiting their bioavailability can protect cells from oxidative-nitrosative stress. The Fenton reaction involves the formation of hydroxyl radicals (·OH) during the catalytic action of divalent metal ions (mainly Fe^2+^) in an aqueous environment [125]. Ergothioneine is characterized by the ability to form strong chelate complexes with copper and iron, preventing them from participating in the Fenton reaction [64]. Likewise, ergothioneine can compete with other metal complexes in chelation of copper ions. This inhibits the formation of protein carbonyl groups, thus protecting DNA and proteins from copper-induced oxidative damage [126,127,128]. On account of its biological effects, ergothioneine has attracted the attention of many researchers as a substance with anti-aging potential. In recent times, ergothioneine supplemented daily at a dose of 4–5 mg/kg/day was shown to promote longevity in male mice and in roundworms (*Caenorhabditis elegans*) [129]. Another study observed the ability of ergothioneine as a modulator of critical aging pathways such as the insulin/IGF, sirtuin 6 (SIRT6) and mammalian target of rapamycin mTOR pathways. Ergothioneine promoted cellular longevity and ameliorated age-related disorders by increasing SIRT6, involved in DNA repair, and inhibiting the mammalian target of rapamycin, mTOR, associated with metabolic dysregulation [130]. Interestingly, the observed mechanisms underlie the longevity phenomenon and alleviation of frailty symptoms observed in a previous study. The dual function of ergothioneine as a direct antioxidant and regulator of the aging process suggests that it may provide effective protection against oxidative damage to sensitive and aging-prone tissues, such as the skin. Indeed, the well-documented anti-aging potential of ergothioneine has made it an attractive target for research on skin aging.

#### 2.1.4. Skin Effects of Ergothioneine

In recent years, evidence has emerged that ergothioneine is a promising dermoprotectant. While the antiaging activity of ergothioneine is not fully understood, the results of one large study suggest that ergothioneine may exert dermoprotective effects by counteracting the cellular accumulation of AGEs. Ergothioneine was observed to inhibit AGE production in a concentration-dependent manner in human dermal fibroblasts (HDFs) exposed to glyceraldehyde. Furthermore, in the same study, Western blot analysis demonstrated a dose-dependent decrease in RAGE expression in ergothioneine-treated and glyoxal-stimulated HDF cells [131].

Ergothioneine attenuates UVA-induced skin aging. Hseu et al. demonstrated that ergothioneine inhibited UVA-induced ROS production in human dermal fibroblasts (HSFs) cells in vitro. A dermatoprotective effect was achieved already at submicromolar concentrations of ergothioneine (0.125–0.5 µM) [132]. Similar effects were observed in an in vitro model of human keratinocytes exposed to UVA radiation, where even nanomolar concentrations of ergothioneine protected epidermal cells from excessive ROS generation [118]. Interestingly, Dong et al. observed that ergothioneine is significantly faster and more effective than coenzyme Q_10_ in directly scavenging free radicals and protecting cells from ROS [102]. The data presented by Obayashi et al. indicate that the antioxidant effect of ergothioneine in skin cells exposed to UV radiation may result directly from the removal of superoxide anion radicals and singlet oxygen generated through photosensitization [110]. Hseu et al. demonstrated that ergothioneine protects human keratinocytes from UVA-induced ROS generation, DNA strand breaks, as well as mitochondrial dysfunction and apoptosis [118]. The action of ergothioneine was closely related to the activation of cellular antioxidant genes via Nrf2/ARE. Ergothioneine acts as an activator of the transcription factor Nrf2 to enhance endogenous antioxidant mechanisms [118,133,134,135]. Nrf2 is a member of the Cap-N-Collar family of transcription factors and recognizes an antioxidant response element (ARE) in the promoter of target genes [136]. At steady state, Nrf2 binds to Kelch-like ECH-associated protein 1 (Keap1) in the cytoplasm through interactions between the Nrf2 moiety and the Keap1 dimer [137]. Under conditions of oxidative stress, Nrf2 dissociates from Keap1, protecting Nrf2 from proteasomal degradation and allowing its entry into the cell nucleus. Stress factors include both endogenous activators, such as ROS and RNS, and exogenous factors, such as heavy metals and xenobiotics and their metabolites [138]. There are two distinct mechanisms responsible for the dissociation of Nrf2 from Keap1. The first is where reactive cysteines belonging to Keap1 form protein–protein cross-links upon reaction with electrophiles, leading to disruption of the Keap1-Nrf2 interaction and release of Nrf2 [139]. The second mechanism involves secondary sensor proteins and activation of protein kinase signaling pathways, resulting in Nrf2 phosphorylation, increased stability, and/or release of Nrf2 from Keap1 [140]. Upon entering the nucleus, Nrf2 forms dimers with small Maf proteins, leading to Nrf2 binding to AREs present in the promoter of Nrf2 target genes and transcriptional activation of these genes, which are *HO-1*, *NQO-1*, *and γ-GCLC* [141]. Hseu et al. provided evidence that increased nuclear translocation of Nrf2 and higher ARE activity underlie the protective effects of ergothioneine on skin. Ergothioneine activated the Nrf2/ARE pathway in human fibroblasts exposed to UVA in a dose- and time-dependent manner, inducing the expression of antioxidant genes (*HO-1*, *NQO-1*, and *γ-GCLC*) and demonstrating dermatoprotective effects in UVA-exposed fibroblasts. In cells with reduced Nrf2 expression, the antioxidant effect was inhibited, indicating that Nrf2 is a key factor for protective effects in the skin. Moreover, ergothioneine inhibited the translocation of the AP-1 complex (c-Fos and c-Jun) in UVA-irradiated fibroblasts, while simultaneously inhibiting MMP-1 activity, which prevented the degradation of type I collagen [132]. Obayashi et al. also observed that ergothioneine treatment of fibroblasts in a UVA model resulted in a nearly 50% reduction in MMP-1 expression, reduced MMP-1 mRNA expression, and further inhibited TNF-α expression at the transcriptional level [110]. Additionally, exposure of HDF cells to UVA increased the content of β-galactosidase, which is associated with aging, compared to the control group. UVA irradiation of HDF cells resulted in a nearly 45% reduction in SA-β-gal concentration at 400 µM ergothioneine compared to HDFs exposed to UV alone [131].

UVB radiation induces skin damage and photoaging through a number of harmful effects, including ROS generation, epidermal cell apoptosis, and inflammation. Ko et al. demonstrated in their in vitro studies using cocultures of UVB-irradiated keratinocytes and fibroblasts that ergothioneine treatment protected keratinocytes from ROS overproduction, maintained the activity of the Nrf2/*HO-1* signaling pathway, and inhibited the activation of proapoptotic proteins. Furthermore, ergothioneine demonstrated anti-inflammatory effects by reducing the concentration of pro-inflammatory cytokines, including IL-1β, IL-6, and TNF-α, while modulating paracrine signaling between keratinocytes and fibroblasts, which led to inhibition of collagen degradation and increased type I procollagen biosynthesis [133]. Ergothioneine also inhibited the increase in TNF-α expression in UVB-irradiated fibroblasts [110]. Notably, in the study by Tsay et al., ergothioneine at a concentration of 1 μM effectively inhibited MMP-1 activity and showed better regeneration of procollagen I, exceeding the activity of ferulic acid and glutathione in human skin fibroblasts irradiated with UVB by inhibiting the translocation of AP-1 (c-Fos and c-Jun complex) [142]. Administration of ergothioneine to mice exposed to UVA and UVB radiation resulted in a decrease in malondialdehyde concentration and an increase in superoxide dismutase activity. Furthermore, there was an increase in PI3K/Akt/Nrf2 signaling in the skin of these animals [143].

Oxidative damage to mitochondria has been recognized as a molecular basis of aging [144]. Bazela et al., by exposing epidermoid carcinoma cell line (KB) cells to UVA and UVB radiation and treating them with 20 µM ergothioneine, demonstrated that ergothioneine regenerates reduced glutathione levels and protects skin cells from mtDNA damage associated with photoaging [145]. Ergothioneine’s role in mtDNA protection may involve combating ROS generated during electron transport [124]. Like skin, hair also undergoes the aging process [146]. Aging of hair follicles manifests as hair loss and graying, and oxidative stress plays a significant role in this process [147]. Hair follicles are embedded deep in the dermis, therefore it is believed that aging-related changes in the surrounding scalp may disrupt the functioning of hair follicles [147,148]. A comprehensive study, including an in vivo mouse model, a dermal papilla cell culture system (DPC), and a hair follicle culture system (HF), examined the anti-aging potential of ergothioneine against hair follicle aging. In vivo experiments, ergothioneine demonstrated excellent results in reducing pigmentation disorders and hair loss in aged mice and in mice with hydrogen peroxide-induced graying. The results of the experiment on DPC cells indicate a mitigating effect of ergothioneine on H_2_O_2_ induced damage. In hair follicle tissue culture, ergothioneine was shown to promote hair growth and pigmentation [149]. Moreover, ergothioneine regulated the proliferation and differentiation of B16-F10 melanocytes exposed to UVA radiation in vitro by reducing the concentration of intracellular ROS [150]. However, the effect of ergothioneine on hair follicle aging remains unclear and requires further research.

Animal studies also provided interesting information. Ergothioneine demonstrated protective properties against X-ray-induced skin radiation damage in vivo. Tan et al. developed a sodium hyaluronate dressing containing 5 mg/mL of ergothioneine and used it to treat X-ray-damaged skin of male BALB/c mice. The dressing demonstrated high effectiveness in scavenging ROS and reducing oxidative stress in vivo, reducing cellular apoptosis and inflammation [151]. Furthermore, ergothioneine administered to chitosan–hyaluronic acid membranes promoted wound healing in an in vivo model of ischemic wounds in rabbit skin. Valachowa et al. observed that the release of ergothioneine from biopolymer membranes enabled its transport to the site of inflammation through the bloodstream via the OCTN1 protein. Furthermore, ergothioneine was more effective than histidine, especially in the early stages of healing [152]. However, the available evidence on the role of ergothioneine in glycooxidation remains limited, despite the fundamental contribution of this process to skin aging. Consequently, its antiglycooxidative potential remains insufficiently characterized, highlighting the need for further research to determine whether ergothioneine may protect the skin against glycooxidative damage and contribute to its potential anti-aging effects.

### 2.2. Ergothioneine in Cosmetics

In recent years, the demand for cosmetics based on natural raw materials has increased significantly. Consumers are increasingly choosing cosmetic products based on natural or organic ingredients, perceived as healthier, safer, and more environmentally friendly. Ergothioneine is a substance that can be successfully incorporated into most cosmetic formulations due to its unique biological effectiveness in vitro and in vivo, high stability, and good water solubility. Unfortunately, there are still no clinical studies confirming the effectiveness of topical skin application of preparations containing ergothioneine. To date, only one clinical trial has been identified that examined the effect of ergothioneine on melanin production in the skin following exposure to UVA radiation. In a clinical study involving 31 volunteers, Liu et al. first induced hyperpigmentation in patients by exposing their skin to UVA radiation and then topically administered a preparation containing 0.1% ergothioneine for 4 weeks. The experiment resulted in a significant reduction in melanin production and skin lightening in the ergothioneine-treated group. Interestingly, this effect was comparable to or better than that of vitamin C [150].

In recent years, ergothioneine has become a common ingredient in various cosmetic formulations. We conducted a review of cosmetics containing ergothioneine available on the market in 2026, subsequently presenting selected preparations in the form of tables (Table 1; the exact composition of the preparations is given in Table S1). We classified the cosmetics according to functional categories: face creams, serums, eye preparations, toners and essences, photoprotective products, and body care products. The list compiled in this table is for illustrative purposes only. The aim of the review was to assess the scope of ergothioneine’s use in modern cosmetic formulations. In order to ensure representativeness, product examples from various market segments and manufacturers were included.

Despite the widespread presence of ergothioneine in various types of preparations, we did not find a preparation in which it served as a single active ingredient. In addition, ergothioneine was rarely found in products as a primary active ingredient. In most cosmetic formulations, ergothioneine appeared as a supporting ingredient. Interestingly, in each preparation, it served solely as an antioxidant. There is an evident information gap regarding the optimal dosage of ergothioneine in cosmetic products. Even though its systemic bioactivity has been characterized to some extent, data on percutaneous penetration are still insufficient. Meanwhile, only a few products available on the market declare the percentage of ergothioneine in the formulation. Thus, the available data are insufficient to objectively compare available preparations and assess their potential effectiveness. In addition, there are no clinical studies confirming the effectiveness of topical ergothioneine. Clinical studies would be helpful in standardizing optimal application parameters, such as concentration, formulation form, and required application time, which would determine the maximum effectiveness of ergothioneine on the skin. Another aspect is the insufficient number of head-to-head studies comparing the effectiveness of ergothioneine to vitamin C, vitamin E, or coenzyme Q10, despite their synergy being found in cosmetics. Ergothioneine appears primarily as an antioxidant in cosmetics, which clearly indicates the need for further research aimed at understanding new mechanisms of action of this compound in the skin.

### 2.3. Dietary Sources of Ergothioneine

**Figure 3:**
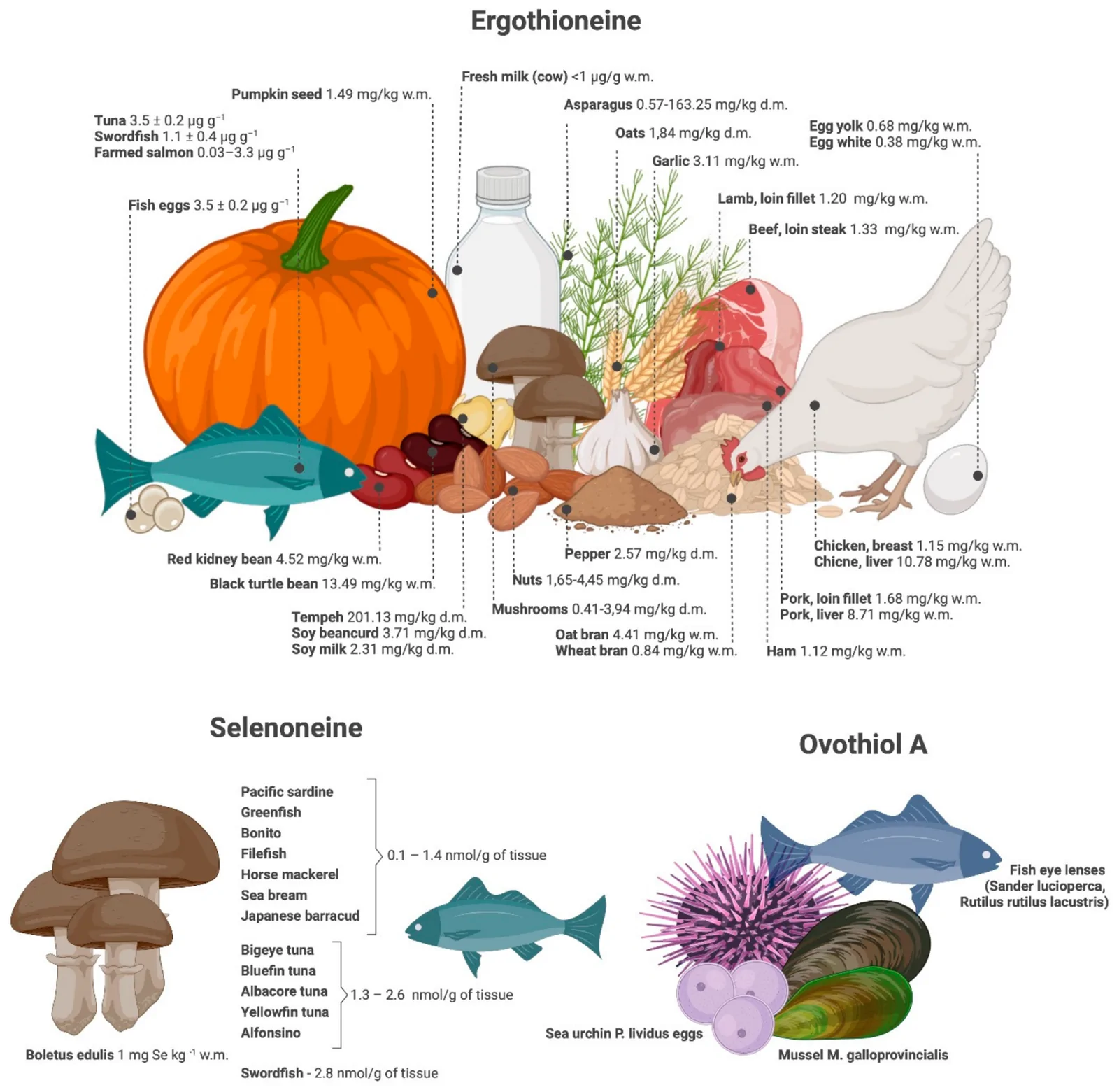
Dietary sources of ergothioneine, selenoneine, and ovothiol. Created in BioRender. Maciejczyk, M. (2026) https://BioRender.com/jiu08jf.

The content of ergothioneine in food products is quite variable [75,87] (Figure 3). The main source of ergothioneine in the human diet is edible mushrooms [153,154]. It is therefore not surprising that ergothioneine has been proposed as a biomarker of mushroom consumption in humans [155,156]. Ergothioneine content can vary significantly depending on the species of mushroom [153,157]. According to available data, lemon oyster mushrooms (*Pleurotus citrinopileatus*, 3.94 mg/g d.m.) [154] and gray oyster mushrooms (*Pleurotus ostreatus*, approximately 1.21 mg/g d.m.) [154] are characterized by high ergothioneine content. Another mushroom rich in ergothioneine is the shiitake mushroom (*Lentinula edodes*, approximately 0.92 mg/g d.m.) [154]. However, the leader in ergothioneine content among all mushrooms is the boletus (*Boletus edulis*, 7.27 mg/g d.m.) [154]. The common mushroom (*Agaricus bisporus*) is also worth mentioning, which, despite its relatively low ergothioneine content (approx. 0.41 mg/g d.m.), is frequently consumed and may be an important dietary source of ergothioneine [158]. Small amounts of ergothioneine have also been found in grains, soybeans, garlic, red beans, asparagus, Brazil nuts, pumpkin seeds, fresh milk, and some spices [75,87,154,159]. Notably, recent studies have also identified ergothioneine in the muscle tissue of fish, such as *tuna* (3.5 ± 0.2 μg g^−1^) and *swordfish* (1.1 ± 0.4 μg g^−1^), as well as *farmed salmon* (0.03–3.3 μg g^−1^), which constitute a significant part of the diet [160]. Ergothioneine has also been detected in fish eggs, and its content was within the range of values reported for tuna [38]. Ergothioneine was previously difficult to obtain, but today, thanks to advances in biotechnology, its efficient biosynthesis is possible [161]. In its pure form, this compound occurs as a white, fine, odorless powder with a sweet taste [162].

### 2.4. Dietary Supplements with Ergothioneine

Ergothioneine has been thoroughly tested for safety and has been determined safe for use as a food additive and dietary supplement by the European Food Safety Authority (EFSA) [83]. EFSA determined the safe daily intake of ergothioneine to be 30 mg/day for adults and 20 mg/day for children aged 3–17 years [83]. A year later, a correction was issued specifying the safe daily intake for pregnant and breastfeeding women (1.31 mg/kg bw/day), children aged 1–3 years (3.39 mg/kg bw/day), and infants (up to 1 year of age) (2.28 mg/kg bw/day) [163]. It should be noted, however, that reference intake values for ergothioneine have not been established. On the other hand, the U.S. Food and Drug Administration (FDA) issued a declaration granting ergothioneine Generally Recognized as Safe status and recommended the use of 5 mg/serving of ergothioneine in food additives [164]. The above findings are based on a comprehensive analysis of toxicological data, metabolic studies, nutritional impact and allergenicity assessment of ergothioneine, the results of which do not indicate a significant risk at the proposed intake doses [83,163,164].

Clinical trial results to date suggest that ergothioneine supplementation at doses up to 25 mg/day is safe and well-tolerated. Cheah et al. conducted the first pharmacokinetic study, demonstrating that administration of pure ergothioneine to healthy young men at doses of 5 mg or 25 mg for one week resulted in rapid absorption, high retention, and minimal urinary excretion of ergothioneine. Supplementation at both doses resulted in a dose-dependent increase in ergothioneine concentrations in blood. Plasma ergothioneine concentrations remained elevated for four weeks after discontinuing supplementation, demonstrating its high bioavailability, stability, and potential renal reabsorption, distinguishing ergothioneine from many amino acids and water-soluble vitamins [84]. In a small (*n* = 19), double-blind, randomized, placebo-controlled study, Yau et al. observed that ergothioneine supplementation at a dose of 25 mg three times a week for one year improved cognitive function in individuals aged 60 years or older, compared to placebo. Moreover, ergothioneine supplementation was shown to be safe, as it did not affect blood counts or liver or kidney function during the study [165]. The results of a much larger, randomized, double-blind, placebo-controlled study by Zajac et al. showed that ergothioneine supplementation at a dose of 10 mg or 25 mg daily in adults aged 55–79 years (*n* = 147) resulted in improved prospective memory and sleep initiation in a dose-dependent manner, with ergothioneine at a dose of 25 mg/day performing significantly better. Furthermore, ergothioneine supplementation improved liver function and was also responsible for increasing telomere length [166]. Another study demonstrated that ergothioneine may alleviate sleep problems through a multifaceted mechanism of action. Katsube et al., in their randomized, double-blind, placebo-controlled study, demonstrated that oral administration of ergothioneine at a dose of 20 mg daily for 4 weeks resulted in improved sleep onset. Electroencephalographic measurements showed that ergothioneine prolonged N2 while shortening N1 during NREM sleep. Meanwhile, the frequency of awakenings after sleep onset was reduced compared to the placebo group. Remarkably, ergothioneine supplementation resulted in reduced consumption of antioxidant and anti-inflammatory substances, lower serum glutamate levels, and improved lipid metabolism in the study patients [167]. In a recent exploratory, placebo-controlled study, Okamura et al. observed that even lower doses of ergothioneine, 8 mg/day, significantly improved subjective sleep outcomes. Improvements in factor IV (refreshment) and factor V (sleep duration) of the Oguri–Shirakawa–Azumi scale were noted compared to placebo [168]. However, the role of ergothioneine supplementation in modulating skin function remains unclear.

To our knowledge, Hanayama et al. conducted the first and only evaluation of the effects of oral consumption of an ergothioneine-rich strain of *Pleurotus* species (hiratake) on skin condition [169]. Eighty healthy women were enrolled in this 12-week, randomized, double-blind, placebo-controlled study and randomly divided into two groups: a study group (hiratake) and a control group (placebo). Women in the study group supplemented with hiratake tablets at a dose of 25 mg of ergothioneine per day for 12 weeks. The results clearly indicated that oral administration of ergothioneine-rich hiratake can improve skin hydration. After 12 weeks of supplementation, improvements in the appearance of wrinkles and overall skin texture were observed in the study group compared to the control group. Remarkably, plasma ergothioneine concentrations in the hiratake group were 4.7-fold higher than baseline values (ranging from 3.4 to 15.9 µM); however, plasma ergothioneine concentrations significantly correlated with improved skin moisture and reduced transepidermal water loss (TEWL) in the upper arm [169].

In response to the growing interest in ergothioneine as a potential anti-aging ingredient, it has been introduced into dietary supplements by numerous manufacturers. In the supplement market, ergothioneine is primarily available in capsule and tablet form. We have compiled a list of preparations in which ergothioneine is the only active ingredient (Table 2). This approach allowed for a reliable comparison of the declared value of the substance by manufacturers, without the influence of other ingredients present in multi-ingredient preparations.

### 2.5. Selenoneine

Selenoneine (2-selenyl-N^α^, N^α^,N^α^-trimethyl-L-histidine) is a selenium analogue of ergothioneine in which the sulphur atom at the C2 position of the imidazole ring has been replaced by a selenium atom [39]. Selenoneine exhibits a rare selenol-to-selenone tautomerism; at physiological pH, the selenone form predominates [170,171]. As a result, selenoneine exhibits not only unique redox properties but also promising biological potential, including both classical antioxidant activity and interactions with metal ions [171]. Although many of the chemical properties of selenoneine are still unknown, there is no doubt that the substitution of sulphur atom with selenium in proteins and nucleic acids confers specific properties, making selenoneine an interesting research subject [172].

#### 2.5.1. Selenoneine Biosynthesis

Selenoneine biosynthesis is not a separate metabolic pathway, but a variant of the ergothioneine biosynthesis pathway, carried out in the presence of selenium [71]. The initial steps are identical and include the methylation of histidine to hercynin, also catalyzed by Egt1. The key difference lies in the use of selenocysteine instead of cysteine as the substrate in the conjugation reaction catalyzed by Egt1. This step results in the formation of hercynylselenocysteine, characteristic of selenoneine. However, unlike the ergothioneine pathway, there is no oxidation step to the selenooxide form. The selenocysteine amino acid residue is then removed in a process catalyzed by Egt2, leading to the formation of selenoneine as the final reaction product. Both biosynthetic pathways utilize the same set of enzymes, and the structural differences between ergothioneine and selenoneine result solely from the substitution of a sulphur atom for selenium during the amino acid conjugation step [71]. The minor structural differences between the compounds and the higher free radical scavenging capacity of selenoneine suggest that its advantage stems from the presence of a selenium atom in place of the sulphur. Selenium is an element essential for human health, playing an important role in maintaining redox homeostasis [51,173,174,175]. It is widely distributed in foods such as meat, fish, and nuts [176,177,178,179]. Other dietary sources of selenium include selenocysteine and selenomethionine derivatives found in fruits and vegetables, as well as the selenocystine dimer found in meat [171]. These selenoamino acids can also be incorporated into proteins [180,181,182].

#### 2.5.2. Selenoneine Bioavailability from Dietary Sources

Selenoneine was first isolated from the blood and other tissues of bluefin tuna, *Thunnus orientalis*, and was spectroscopically characterized as a selenoneine dimer, as the monomer was considered too unstable to isolate [183,184]. Reduction of the selenoneine dimer to the monomer was achieved using glutathione or dithiothreitol [184]. Reports suggest that consuming foods rich in selenoneine causes its accumulation in the body. Selenoneine is also absorbed into cells via the ergothioneine transporter OCTN1 [185,186]. It can therefore be assumed that selenoneine will be absorbed by tissues expressing OCTN1 and will accumulate in a manner analogous to ergothioneine [81]. Interestingly, OCTN1 not only participates in the absorption and excretion of selenoneine but also participates in the transport of the selenoneine-MeHg complex, which reduces the toxicity of methylmercury [186]. Nevertheless, the absorption and distribution of selenoneine in in vivo models is not fully understood.

#### 2.5.3. Anti-Aging Properties of Selenoneine

Selenoneine, due to the rare phenomenon of selenol-to-selenone tautomerism, exhibits unique redox properties [170,171]. Electron spin resonance spectrometry results presented by Seko et al. showed that selenoneine, both in its monomeric and dimeric forms, directly scavenged ·OH generated upon exposure to H_2_O_2_ and UV light. The obtained data indicate that selenoneine captures ·OH by autoxidation and subsequent reduction back to the parent molecule. Notably, the same study demonstrated that the free radical scavenging capacity was stronger for the selenoneine monomer than for the selenoneine dimer, with ergothioneine having the lowest efficiency [39]. Moreover, selenoneine demonstrated significantly stronger ROS scavenging activity than ergothioneine in the in vitro DPPH assay [183]. Therefore, selenoneine may be an important member of the redox cycle in animal cells. Promising biological properties of selenoneine include both classical antioxidant activity and interaction with metal ions. Selenoneine mitigates MeHg accumulation and associated toxicity [184]. Yamashita et al. provided evidence that selenoneine accelerates OCTN1-mediated excretion and demethylation of methylmercury in an in vitro model of human embryonic kidney cells (HEK293) and zebrafish embryos. After exposure of HEK293 cells and embryos to MeHg-cysteine, MeHg accumulation was reduced, and MeHg excretion and demethylation were enhanced by selenoneine [186]. Providing selenoneine through fish consumption may reduce the formation of ROS and thus inhibit the aging process [184]. Tohfuku et al., using an in vivo model of fish fed a diet containing selenoneine, demonstrated that dietary selenoneine administration led to its accumulation in the blood and muscles of giant amberjacks, resulting in a reduced oxidation-reduction potential and decreased ROS production [187]. Furthermore, a study by Rohn et al. reported that selenoneine administration in *Caenorhabditis elegans* led to a reduction in the formation of ROS and RNS [188].

Selenoneine may be a dietary source of selenium [183]. Selenium-containing compounds are of particular interest due to the relatively rare occurrence in nature and a number of beneficial biological properties [46,47,175,176,177,178,179,182]. Data on the biological properties of selenoneine and its importance for human health are still incomplete. However, Drobyshev et al. revealed that selenoneine can cross the blood–brain barrier in vitro [189]. Given the structural analogy of ergothioneine, it can be assumed that selenoneine may exhibit similar properties [190]. Hanafi et al. demonstrated that selenoneine is the predominant form of selenium in all seabird tissues examined, with its content in brain tissue being 78–88% [191]. Experimental data suggest that selenoneine may play a key role in protecting the nervous system from oxidative stress [192,193,194]. However, the number of studies on selenoneine remains limited, and its full anti-aging potential is still not well understood. In particular, its antiglycooxidative potential remains largely unknown, despite the fundamental role of glycooxidation in the skin aging process. Further studies are therefore needed to determine whether selenoneine may exert protective effects against this process and contribute to its potential anti-aging activity.

#### 2.5.4. Skin Effects of Selenoneine

Currently, the effects of selenoneine on the skin remain poorly understood. There is a lack of experimental data evaluating its effects on skin cells, as well as studies investigating its potential biological activity in the skin. In particular, the mechanisms underlying its possible antiglycooxidative effects have not yet been fully characterized. Furthermore, it remains unknown whether selenoneine can penetrate the epidermis and reach the dermis following topical application, or whether the OCTN1 transporter is involved in its transepidermal transport. These significant research gaps highlight the need for further in vitro and in vivo studies to elucidate the potential role of selenoneine in skin protection and anti-aging processes.

#### 2.5.5. Dietary Sources of Selenoneine

Selenoneine has been shown to be found primarily in certain types of marine fish. A study by Yamashita et al. demonstrated that the highest concentrations of selenoneine are found in *swordfish* muscles (2.8 nmol/g of tissue) [195]. In turn, the selenoneine content in the muscles of *bigeye tuna*, *bluefin tuna*, *albacore tuna*, *yellowfin tuna* and *alfonsino* was nearly 1.3–2.6 nmol/g of tissue [195]. Furthermore, in other fish species, such as *Pacific sardine*, *greenfish*, *bonito*, *filefish*, *horse mackerel*, *sea bream*, and *Japanese barracuda*, muscle selenoneine concentrations ranged from 0.1 to 1.4 nmol/g of tissue [195]. To date, marine fish have been the only significant nutritional source of selenoneine consumed by a significant portion of the global population [184,196,197,198,199,200]. However, it has recently been shown that the porcini mushroom (*Boletus edulis*) is a richer source of selenoneine than commonly consumed marine fish such as tuna and mackerel [201]. Regardless of the above observations, further research is necessary to confirm and expand our understanding of natural sources of selenoneine in food raw materials. Despite the growing interest and evidence for the presence of selenoneine in food, there is still a significant gap in data regarding safe intake levels and reference values for selenoneine. Current data indicate no toxic effects of selenoneine in humans, but biological evaluation appears limited because selenoneine is not commercially available [171]. Due to its biological activity, selenoneine is being considered as a potential ingredient in cosmetic and nutricosmetic preparations with anti-aging properties. Despite its presence in the diet, available data on the safety profile and systemic effects of selenoneine on human health remain limited, making it difficult to assess the validity of its use as dietary supplements. Similarly, the use of selenoneine in cosmetic preparations requires further research due to insufficient data on its effects on skin cells. Consequently, the potential for selenoneine’s anti-aging applications has not yet been clearly established.

### 2.6. Ovothiol A

Ovothiol A (1-methyl-4-thiol-L-histidine) is a naturally occurring histidine derivative in which the hydrogen atom at the C5 position of the imidazole ring has been replaced with a sulphur atom [40,41]. Ovothiol A belongs to the family of three mercaptohistidines (ovothiols: A, B, and C), compounds abundantly found in the egg cells and ovaries of marine invertebrates [202]. In vitro studies on the redox chemistry of ovothiols indicate their exceptionally high protective potential against oxidative stress [203,204] and peroxynitrite-induced damage [205]. Furthermore, experimental data provided by Turner et al. indicate that the activity of ovothiols is remarkably similar to that of glutathione peroxidase (GPx) [206]. The set of properties mentioned suggests a protective role of ovothiols under environmental stress conditions, which is in agreement with previous hypotheses by Shapiro et al. on the potential protective role of ovothiols against ROS generated by the respiratory burst induced by fertilization in marine organisms [207]. In this context, ovothiol A demonstrates particular ROS scavenging activity. The -SH group of ovothiol A has a very low pKa value (pKa ≈ 1.0–1.4) [41,208,209], compared to other natural thiols, such as glutathione (pKa = 8.7) [55,203,208,209,210,211]. Therefore, ovothiol A occurs almost exclusively in the thiolate form, a feature that underlies its broad spectrum of biological effects.

#### 2.6.1. Biosynthesis of Ovothiol A

Ovothiol A is synthesized via similar enzymatic reactions to ergothioneine. In the initial stage of biosynthesis, a C-S bond is formed between the sulfhydryl group of cysteine and C-5 of the imidazole ring of histidine in a reaction catalyzed by sulfoxide synthase (OvoA (homolog of EgtB and Egt1)) in the presence of oxygen and iron [212,213,214]. In the second stage, removal of the cysteine residue by OvoB (a PLP-dependent lyase) results in the formation of 5-thiohistidine, the direct precursor of ovothiol A, and the release of pyruvate and ammonia. Termination of the ovothiol A biosynthetic pathway involves OvoA-catalyzed methylation of the π-nitrogen of the imidazole ring of histidine, yielding the final product [212,214,215]. Ovothiol A is considered one of the most potent natural antioxidants, and recent reports describe ovoselenol, a derivative of ovothiol A, as the most recent discovery in this family of compounds. Ovoselenol is a selenium analog of ovothiol A in which the sulphur atom attached to C-5 of the imidazole ring of histidine has been replaced by a selenium atom [216]. Although ovothiol A exhibits significant antioxidant properties, recent reports on ovothiol A suggest the possibility of different redox properties resulting from the presence of the selenium atom [216,217,218].

#### 2.6.2. Ovothiol A Bioavailability from Dietary Sources

Ovothiol A was first isolated from the eggs of marine invertebrates [219,220,221,222,223] and was later found in other species, including marine algae [224,225,226], and intracellular pathogens [227]. Available data on its chemical nature suggest that ovothiol A may be absorbed by animals in a manner analogous to ergothioneine and selenoneine [211,212,213,214,215,216,217,218,219,220,221,222,223,224,225,226,227,228], but this area requires further research.

#### 2.6.3. Anti-Aging Properties of Ovothiol A

Ovothiol A possesses strong antioxidant properties due to the specific position of the sulfhydryl group on the imidazole ring of histidine, providing unique reactivity towards ROS [41,212,217,229]. The pKa value of the -SH group of ovothiol A is significantly lower (pKa ~1.4) compared to other natural thiols, such as glutathione (pKa ~8.7), cysteine (pKa ~8.4), and coenzyme A (pKa ~9.8) [40,41,208,209,229]. Because ovothiol A has a very low pKa, at physiological pH it exists almost entirely in the thiolate (S-) form. This differs from glutathione, which has a high pKa and, under such conditions, remains largely undissociated. Therefore, ovothiol A is a much more effective antioxidant because, compared to glutathione, it reacts much more quickly with oxidants (Fremy’s salt, ferricytochrome c, H_2_O_2_, and the tyrosyl radical) [55,202,203,207,210,211]. At the same time, the two-electron redox potential of ovothiol A is significantly higher (−0.09 V) than that of glutathione (−0.26 V) [229]. Early studies suggested that the primary role of ovothiol A is to protect eggs from the harmful effects of hydrogen peroxide generated during the oxidative burst accompanying fertilization [207,219,230]. According to the literature, ovothiol A biosynthesis naturally occurs under extreme conditions, including exposure to heavy metals and toxins, as well as UV radiation [231,232,233]. Oxidative stress is considered one of the main factors contributing to skin aging [95]. In this context, ovothiol A, due to its chemical properties, may be considered a potential candidate for an anti-aging compound [212]. Ovothiol A is considered one of the most powerful antioxidants found in nature [234]. The recent discovery of the presence of ovothiol A in the lens of freshwater fish is therefore not without significance. The critical protective function of ovothiol A against the fish lens may suggest its potential dermatoprotective use [234]. Castellano et al. demonstrated that ovothiol A demonstrated high therapeutic potential against diseases associated with oxidative stress and inflammation in an in vitro cellular model of hyperglycemia-induced endothelial dysfunction [235].

In a study by Brancaccio et al., ovothiol A demonstrated antifibrotic activity in a mouse model of liver fibrosis. The hepatoprotective effect was associated with a reduction in the concentration of fibrogenic markers involved in the liver fibrosis process, such as transforming growth factor-β (TGF-β), α-smooth muscle actin (α-SMA), and tissue inhibitor of metalloproteinases 1 (TIMP-1). It is hypothesized that the attenuation of liver fibrosis with ovothiol A may be regulated by the expression and activity of cellular γ-glutamyl transpeptidase (GGT), which plays a key role in antioxidant defense [236]. More recent research findings expand our understanding of ovothiol A as a natural GGT inhibitor [237]. Both the aging process and thyroid disease are strongly associated with increased oxidative stress, which leads to cellular damage and impaired tissue function [95,238]. Gaber et al. demonstrated that supplementation with ovothiol A in rats with thyroxine-induced hyperthyroidism resulted in a significant reduction in nitric oxide and TNF-α levels, as well as significant improvement in thyroid and liver structure and a significant reduction in collagen deposition. These protective and therapeutic effects of ovothiol A were achieved by stimulating the antioxidant system, reducing inflammation, and restoring normal thyroid hormone levels [239]. The pathophysiological processes observed in peptic ulcer disease and skin aging exhibit significant molecular similarities; in both cases, chronic inflammation and oxidative stress play a key role, leading to increased pro-inflammatory cytokines, pro-inflammatory pathway activity, and increased MMP activity [240,241]. Salaheldin et al., in an in vivo rat model of gastric ulcer disease, observed that ovothiol A reduced levels of malondialdehyde (MDA), interleukin-6 (IL-6), and cytochrome c, and also contributed to the increase in key components of the antioxidant defense system, such as glutathione (GSH), catalase (CAT), glutathione-S-transferase (GST), superoxide dismutase (SOD), and nitric oxide (NO). Furthermore, an improvement in gastric tissue structure was observed. The antiulcer effect was determined by free radical scavenging, inhibition of inflammation, regulation of apoptosis, and stabilization of fibroblast growth [242]. The unique antioxidant effect of ovothiol A was also confirmed by Mohammed et al. in an in vivo rat model of myocardial infarction. Ovothiol A treatment restored normal function and improved cardiac biomarker profiles [243].

#### 2.6.4. Skin Effects of Ovothiol A

Despite the growing interest in ovothiol A, the number of studies assessing its effect on skin homeostasis remains relatively small. Brancaccio et al. conducted the first and, at the time, only comprehensive analysis of the effect of ovothiol A on IL-1β-induced inflammation in human skin cells and tissues. They observed that ovothiol promoted Nrf2 accumulation in the nuclei of HaCaT keratinocytes. Furthermore, a decrease in *IL-8*, *TNF-α*, and *COX-2* gene expression was observed in human skin tissues, along with a significant decrease in the production of the cytokines IL-6, IL-8, and TNF-α. Interestingly, the protective effect of ovothiol A was stronger than dexamethasone, a corticosteroid currently used to treat inflammatory skin conditions [244]. One of the main factors contributing to premature skin aging and the risk of skin cancer development is UV radiation. Luccarini et al. performed spectrophotometric and fluorometric analysis of two ovothiol derivatives: 5-thiohistidine and iso-ovothiol A, regarding UVA absorption, photostability, and UVA protection in vitro. The results of this study are clear and indicate that these compounds may represent an alternative approach to skin protection from UVA radiation. It is postulated that their activity could reduce oxidative damage to proteins and lipids through excellent absorption of the UVA spectrum. The compound’s ability to absorb UVA radiation may determine its potential photoprotective properties by limiting radiation penetration into the deeper layers of the skin [245]. Ovothiol A could be used to develop natural skin care products. The promising action profile of ovothiol A indicates its potential use in broad-spectrum formulations, including anti-inflammatory preparations aimed at reducing inflammation induced by environmental stressors, as well as *anti-aging* products and photoprotective preparations. The number of studies is limited, and the full anti-aging potential of ovothiol A remains largely unexplored. A particularly important research gap is the lack of studies investigating the antiglycooxidative potential of ovothiol A, despite the fundamental role of glycooxidation in the skin aging process. Due to its origin, ovothiol A fits into the current search for innovative, natural active ingredients of marine origin. Although the biological potential of ovothiol A makes it a promising ingredient in cosmetic preparations, such products have not yet been developed. However, the high instability of ovothiol A constitutes a significant technological challenge in the development and production of cosmetic formulations [234].

#### 2.6.5. Dietary Sources of Ovothiol A

The main known sources of ovothiol A among marine invertebrates are the sea urchin *P. lividus* and the mussel *M. galloprovincialis* [42]. Sea urchin eggs, which contain ovothiol in millimolar concentrations [220,222], are the source most commonly used to obtain pure ovothiol A. However, recently, the accumulation of ovothiol A in high concentrations in the mantle of female mussels at stage 5 of oogenesis has also been reported [246]. More recently, ovothiol has also been detected in other tissues, including the gills and lenses of freshwater fish, suggesting a broader tissue distribution than previously recognized [247,248,249]. These findings indicate that ovothiol may be a much more widespread metabolite in both freshwater and marine animals than previously thought and may play an important role in antioxidant defense across various tissues and species. Although both of these marine raw materials are edible, no data are available on the absorption, recommended doses, or safety of ovothiol A from the diet. The wide range of biological properties of ovothiol A suggests its potential use as a dietary supplement. However, the lack of available data on the safety profile of ovothiol A and the lack of detailed studies assessing the systemic effects of ovothiol A on the human body prevent confirmation of such use.

### 2.7. Conclusions and Future Perspectives

Ergothioneine, selenoneine, and ovothiol A are among the increasingly intensively studied substances of marine origin. Their structure and chemical properties have been well characterized, and the mechanisms of action are gradually being elucidated. All three compounds have been shown to play an important role as regulators of oxidative stress.

Cosmetology is constantly searching for substances with anti-aging properties that are both naturally derived and have a high safety profile. To date, only ergothioneine has been used in cosmetics and dietary supplements due to its high stability, safety of use, and well-documented anti-aging effects.

Despite growing interest in these compounds, the number of clinical studies on their effects on the skin remains limited. In vitro studies on ovothiol A are scarce, while no such data exist for selenoneine. In this paper, we identify significant research gaps and propose directions for future research addressing key, still unresolved issues.

The rate of percutaneous permeation of ergothioneine and selenoneine through the epidermis and whether these compounds are capable of penetrating the dermis have not yet been determined. It also remains unclear whether the OCTN1 transporter participates in the transepidermal transport of these substances and whether its endogenous expression in the skin could increase their biological effectiveness. In the case of ovothiol A, its distribution in the body has not yet been determined. The impact of ergothioneine, selenoneine, and ovothiol A deficiency and excess on human health also remains unexplained. The molecular mechanisms responsible for their antiglycooxidative effects have not yet been fully characterized. Clinical studies evaluating the effects of topical ergothioneine application to the skin are also lacking. The limited number of comparative (head-to-head) studies comparing its activity with commonly used antioxidants makes it difficult to clearly assess its effectiveness. There are no studies comparing selenoneine and ovothiol A with other compounds with anti-aging properties.

**Figure 4:**
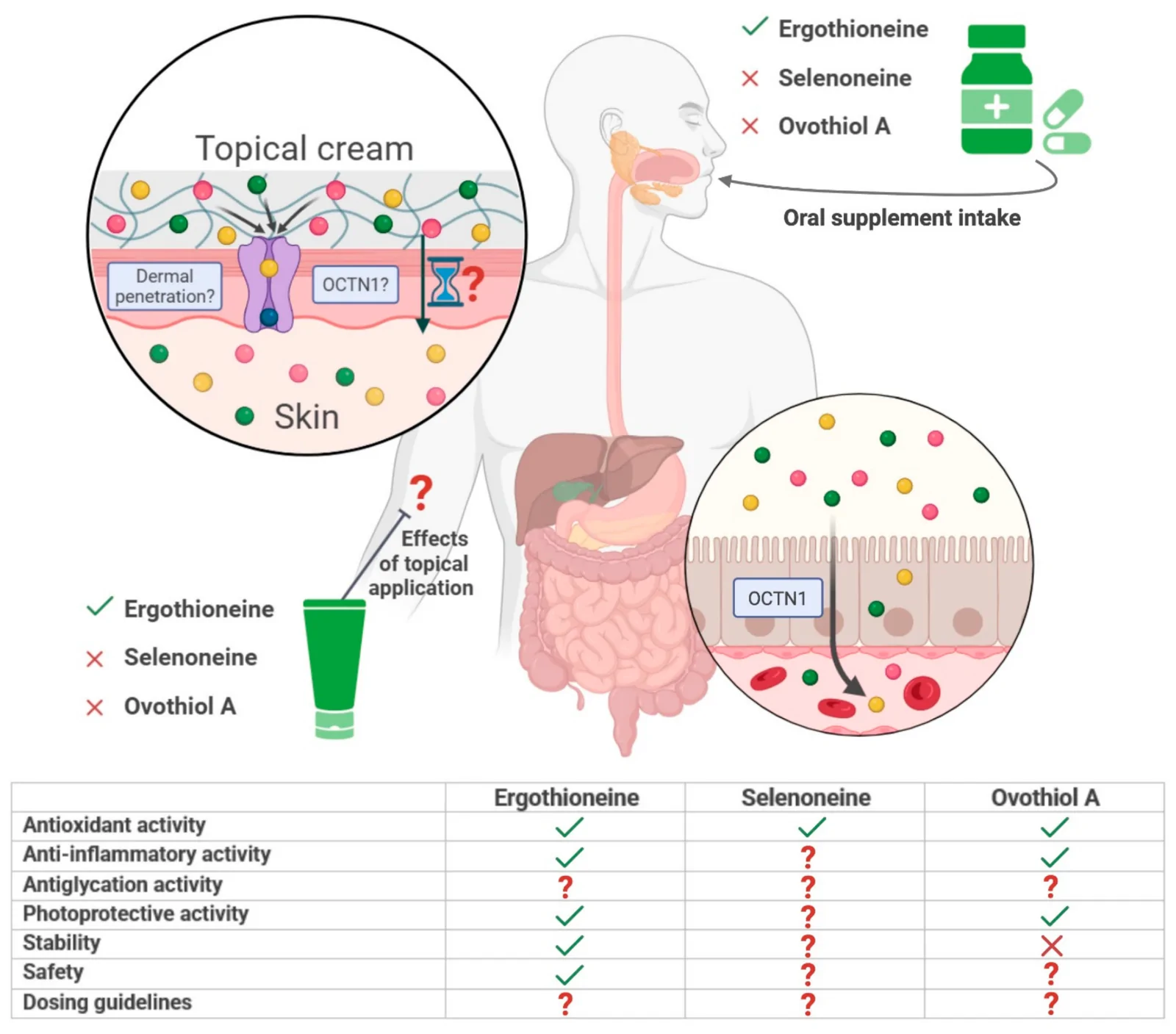
Schematic overview of current knowledge and research gaps related to the effects of ergothioneine, selenoneine and ovothiol A following oral administration and dermal exposure. Created in BioRender. Maciejczyk, M. (2026) https://BioRender.com/ehha6ke.

There is an urgent need to conduct in vitro and in vivo studies on the effects of ovothiol A on skin cells, as well as any studies examining the effects of selenoneine on the skin (Figure 4).

## Figures and Tables

**Table 1:** Summary of commercially available cosmetics containing ergothioneine in 2026.

Product Type	Product	Declared Cosmetic Functions and Effects	Source (Accessed on 15 March 2026)
Face Creams	Estée Lauder DayWear Matte Oil-Control Anti-Oxidant Moisture Gel Crème	Counteracts visible signs of aging and adds vitality and freshness to tired skin.	https://www.esteelauder.pl/
	Kate Somerville Kate Somerville Age Arrest Anti-Wrinkle Cream	Seeks to restore the skin’s bounce by delivering immediate, long-lasting plumping hydration.	https://www.katesomerville.co.uk/
	Clinique Smart Night™ Custom-Repair Moisturizer	Moisturising and anti-aging.	https://www.clinique.com.pl/
	DR. JETSKE ULTEE Moisturizer normal to dry skin	Hydrates, nourishes, and repairs the skin.	https://www.dr-jetskeultee.com/
	REVOLVE High Performance Face Cream	Balancing and protective; provides anti-aging benefits.	https://www.revolve.com/
	Epionce MelanoLyte Tx Brightening cream	Helps address discolouration, dryness, and dullness.	https://www.epionce.pl/
Serums	BasicLab Esteticus Antioxidant Strengthening Serum 0.5% Ergothioneine, 5% Gallic Acid Diglucoside Complex, EGCG—Radiance and Elasticity Day/Night	Promotes firmer, more elastic skin; neutralises free radicals; evens skin tone; reduces the tendency towards discolouration; brightens the complexion; strengthens the epidermal barrier; reduces irritation and redness; limits transepidermal water loss; and leaves the skin moisturised and healthy-looking.	https://basiclab.shop/
	BasicLab Esteticus Antioxidant Strengthening Serum 1% Ergothioneine, 10% Complex	Promotes firmer, more elastic skin; minimises wrinkles and fine lines; neutralises free radicals; evens skin tone; reduces the tendency towards discolouration; brightens the complexion; strengthens the epidermal barrier; reduces irritation and redness; limits transepidermal water loss; and leaves the skin moisturised and healthy-looking.	https://basiclab.shop/
	Photozyme Dna Youth Recovery Facial Serum	Hydrates the skin, neutralises free radicals, and improves overall tone and texture.	https://photozyme.com/
	DoSe By VH Azelaic Acid 10% Serum	Helps achieve clear, bright, congestion-free skin.	https://victoriahealth.com/
	NuFACE NuFACE Protect and Tighten Super Antioxidant Booster Serum	Protects the skin.	https://www.mynuface.com/
	Paula’s Choice 25% Vitamin C + Glutathione Clinical Serum	Improves skin tone, lightens discolouration, and increases skin firmness and elasticity.	https://www.paulaschoice.pl/
Eye cosmetics	DoSe By VH Vitamin C Eye Cream	Visibly reduces the appearance of wrinkles in age-prone areas, minimises under-eye puffiness and dark circles, targets collagen loss to help improve skin texture, and features a precise cooling tip to soothe tired eyes.	https://victoriahealth.com/
	Sesderma Reti Age Eye Contour Gel Eye Cream with Retinol	Leaves the skin around the eyes firmer, smoother, and more radiant.	https://www.sesderma.com/pl
	Dermaquest Peptide 3D Eye Lift	Moisturises and nourishes the skin, reduces irritation and eyelid sagging, smooths wrinkles and fine lines, and leaves the eye area looking fresh and radiant.	https://dermaquest.pl/
	Estée Lauder Re-Nutriv Ultimate Lift Regenerating Youth Eye Crème	Leaves the skin around the eyes firmer, less puffy, stronger, and more resilient to future signs of aging. Minimises lines and visibly reduces dark circles.	https://www.esteelauder.pl/
	grace & stella Brightening eye stick	Brightening, moisturising, and toning.	https://www.graceandstella.com/
Tonics and essences	Allies of Skin Molecular Saviour Probiotics Treatment Mist	Moisturises, regulates excess sebum, detoxifies, brightens, and protects against external factors.	https://eu.allies.shop/
	SESDERMA REPASKIN DEFENSE Mist	Helps address pollution, photoaging, irritation and sensitivity, and discolouration.	https://www.sesderma.com/
Photoprotectants	AMPLEUR Luxury White W Protect UV+ SPF50 Pa++++	Protects the skin not only from UV rays but also from blue light and environmental stressors that accelerate skin aging.	https://ampleur.id/
	Nevoa Active Optimal Defense | Broad Spectrum SPF 43	Helps defend the skin against sun damage while supporting overall skin health.	https://www.neova.com/
	Prep + Prime Face Protect Lotion SPF 50	Protects the skin against a broad spectrum of UVA and UVB radiation.	https://www.maccosmetics.pl/
	Dr Irena Eris BB CREAM SPF 50 Waterproof Tinted Moisturizer	Adapts to the natural skin tone; moisturises, brightens, and evens out the complexion.	https://www.drirenaeris.com/
	Estée Lauder Perfectionist Pro Multi-Defense Aqua UV Gel SPF 50 with 8 Anti-Oxidants	Provides UVA/UVB protection in an invisible veil, as well as protection against pollution and dryness.	https://www.esteelauder.pl/
	SHISEIDO Urban Environment Mineral Clear Sunscreen SPF 50	Provides broad-spectrum SPF 50 protection against UVA and UVB rays. Clinically proven to improve uneven skin tone and radiance in two weeks, and wrinkles and firmness in four weeks. Provides 12-h hydration and is water-resistant for 40 min.	https://www.shiseido.com/
Body care products	SHIKAI 8 oz Borage Therapy Lotion—Original Formula	Provides fast relief for dry, itchy skin.	https://shikai.com/

**Table 2:** List of dietary supplements available for sale in 2026 containing ergothioneine as a standalone ingredient.

Product	Declared Dose per Serving	Declared Effect	Source (Accessed on 20 May 2026)
Vitafit^®^ Ergothioneine 120 capsules	5 mg	Support for daily diet in a modern form	https://firesnakenutrition.eu/
XYMOGEN^®^ MitoPrime— Fermentation-based L-ergothioneine, 30 capsules	12.5 mg	Antioxidant	https://www.xymogen.com/
Kenay^®^ L-ergothioneine ErgoElite™ 60 veggie capsules	15 mg	Antioxidant	https://kenay.com.pl/
California Gold^®^ Nutrition, Ergothioneine, 90 veggie capsules	25 mg	Antioxidant	https://pl.iherb.com/
ProHealth Longevity^®^ Ergothioneine, 30 capsules		Supports longevity by helping maintain telomere length, combats oxidative stress in the brain to support cognitive function, protects the heart and is associated with reduced cardiovascular mortality.	www.prohealth.com
MOLEQLAR^®^ Ergothioneine Capsules		Supports longevity	https://moleqlar.com/
Life Extension GymBeam^®^ Mega L-ergothioneine, 30 vegetarian capsules		Supports healthy aging	https://www.lifeextension.com/
Double Wood^®^ Ergothioneine Supplement (EGT), 60 Count, Non-GMO, Gluten Free, Vegan Safe	30 mg	May Support Longevity And Healthy Aging	https://doublewoodsupplements.com/
GymBeam^®^ L-Ergothioneine		A longevity supplement	https://gymbeam.pl/
GeneIII^®^ L-Ergothioneine		Supports cognitive health, sleep quality, and natural, sustained energy	https://www.geneiii.com/

## Data Availability

No new data were created or analyzed in this study. Data sharing is not applicable to this article.

## Supplementary Materials

The following supporting information can be downloaded at: https://www.mdpi.com/article/10.3390/antiox15091118/s1, Table S1: INCI of the evaluated preparations.
