# Supplementary material for: Natural Histidine Derivatives—From Basic Research to Potential Applications in Cosmetics and Nutricosmetics
Source: Antioxidants (Basel). 2026 Sep 4;15(9):1118. doi: 10.3390/antiox15091118 (PMC13603875; doi:10.3390/antiox15091118)
Supplement: Supplementary file 1 [file antioxidants-15-01118-s001.zip › antioxidants-4488034-supplementary.pdf]

Table S1. INCI of the evaluated preparations.

Product	INCI	Source
Estée Lauder DayWear Matte Oil-Control Anti-Oxidant Moisture Gel Crème	WATER\AQUA\EAU, ETHYLHEXYL SALICYLATE, BUTYLOCTYL SALICYLATE, POLYESTER-8, BUTYL METHOXYDIBENZOYLMETHANE, PETROLATUM, BUTYLENE GLYCOL, HOMOSALATE, CETYL RICINOLEATE, OCTYLDODECYL MYRISTATE, STEARETH-21, STEARETH-2, CAPRYLIC/CAPRIC/MYRISTIC/STEARIC TRIGLYCERIDE, DI-C12-15 ALKYL FUMARATE, DIMETHICONE, TETRAHEXYLDECYL ASCORBATE, TOCOPHERYL ACETATE, THERMUS THERMOPHILLUS FERMENT, **ERGOTHIONEINE**, ETHYLBISIMINOMETHYLGUAIACOL MANGANESE CHLORIDE, PSIDIUM GUAJAVA (GUAVA) FRUIT EXTRACT, GENTIANA LUTEA (GENTIAN) ROOT EXTRACT, POLYGONUM CUSPIDATUM ROOT EXTRACT, HORDEUM VULGARE (BARLEY) EXTRACT\EXTRAIT D'ORGE, LAMINARIA OCHROLEUCA EXTRACT, TRITICUM VULGARE (WHEAT) GERM EXTRACT, HYDROLYZED RICE BRAN EXTRACT, HYDROLYZED RICE EXTRACT, ARTEMIA EXTRACT, ROSMARINUS OFFICINALIS (ROSEMARY) LEAF EXTRACT, VITIS VINIFERA (GRAPE) SEED EXTRACT, STEARYL ALCOHOL, BUTYROSPERMUM PARKII (SHEA) BUTTER, SORBITAN OLIVATE, CAFFEINE, PENTYLENE GLYCOL, LAURYL PEG-9 POLYDIMETHYLSILOXYETHYL DIMETHICONE, BEHENYL ALCOHOL, PANTETHINE, TRIACONTANYL PVP, ACRYLIC ACID/VP CROSSPOLYMER, ETHYLHEXYLGLYCERIN, GLYCERIN, LINOLEIC ACID, POLYQUATERNIUM-51, CHOLESTEROL, CETEARYL OLIVATE, SQUALANE, SODIUM PCA, UREA, CAPRYLIC/CAPRIC TRIGLYCERIDE, CITRIC ACID, SODIUM HYALURONATE, GLYCYRRHETINIC ACID, TREHALOSE, TRIACETIN, LECITHIN, HYDROGENATED LECITHIN, GLUCOSE, TOCOPHEROL, PALMITOYL HYDROXYPROPYLTRIMONIUM AMYLOPECTIN/GLYCERIN CROSSPOLYMER, CYCLODEXTRIN, NORDIHYDROGUAIARETIC ACID, ASCORBYL TOCOPHERYL MALEATE, SODIUM HYDROXIDE, FRAGRANCE (PARFUM), PENTAERYTHRITYL TETRA-DI-T-BUTYL HYDROXYHYDROCINNAMATE, SODIUM CHLORIDE, DISODIUM EDTA, BHT, POTASSIUM SORBATE, CHLORPHENESIN, PHENOXYETHANOL, BENZYL SALICYLATE, LINALOOL, MICA, YELLOW 5 (CI 19140), BLUE 1 (CI 42090), CHROMIUM HYDROXIDE GREEN (CI 77289)	https://www.esteelauder.pl/
Kate Somerville Kate Somerville Age Arrest Anti-Wrinkle Cream	WATER/AQUA/EAU, NEOPENTYL GLYCOL DIHEPTANOATE, CAPRYLIC/CAPRIC TRIGLYCERIDES, CETEARYL ALCOHOL, COCOGLYCERIDES, PROPANEDIOL, GLYCERIN, CETYL ESTERS, DIMETHICONE, TRIDECYL NEOPENTANOATE, STEARYL	https://www.katesomerville.co.uk/

	ALCOHOL, GLYCERYL STEARATE, **ERGOTHIONEINE**, PANTHENOL, TOCOPHERYL ACETATE, PALMITOYL DIPEPTIDE-10, KAPPAPHYCUS ALVAREZII EXTRACT, TOCOPHEROL, TOCOTRIENOLS, ALOE BARBADENSIS LEAF JUICE, AVENA SATIVA (OAT) BRAN EXTRACT, HYDROLYZED HYALURONIC ACID, ADENOSINE, SEA WHIP EXTRACT, TRIPLEUROSPERMUM MARITIMUM EXTRACT, ROSMARINUS OFFICINALIS (ROSEMARY) LEAF EXTRACT, LAVANDULA ANGUSTIFOLIA (LAVENDER) FLOWER/LEAF/STEM EXTRACT, MENTHA PIPERITA (PEPPERMINT) LEAF EXTRACT, PASSIFLORA INCARNATA FRUIT EXTRACT, ANIBA ROSAEODORA (ROSEWOOD) WOOD EXTRACT, CAMELLIA SINENSIS LEAF EXTRACT, CANANGA ODORATA FLOWER EXTRACT, CUPRESSUS SEMPERVIRENS SEED EXTRACT, FUCUS VESICULOSUS EXTRACT, PRUNUS ARMENIACA (APRICOT) FRUIT EXTRACT, JASMINUM OFFICINALE (JASMINE) FLOWER/LEAF EXTRACT, VANILLA PLANIFOLIA FRUIT EXTRACT, CITRUS AURANTIUM BERGAMIA (BERGAMOT) FRUIT EXTRACT, SANTALUM ALBUM (SANDALWOOD) WOOD EXTRACT, CORIANDRUM SATIVUM (CORIANDER) SEED EXTRACT, CUCUMIS MELO CANTALUPENSIS FRUIT EXTRACT, CUCUMIS SATIVUS (CUCUMBER) FRUIT EXTRACT, PRUNUS PERSICA (PEACH) FRUIT EXTRACT, PYRUS MALUS (APPLE) FRUIT EXTRACT, ROSE EXTRACT, RUBUS IDAEUS (RASPBERRY) FRUIT EXTRACT, TETRAHEXYLDECYL ASCORBATE, CETEARETH-20, PEG-40 STEARATE, SODIUM ACRYLATE/SODIUM ACRYLOYLDIMETHYL TAURATE COPOLYMER, ARACHIDYL ALCOHOL, ELAEIS GUINEENSIS (PALM) OIL, CETYL ALCOHOL, SORBITAN OLEATE, ISOHEXADECANE, XANTHAN GUM, ETHYLHEXYLGLYCERIN, POLYSORBATE 80, DISODIUM EDTA, POTASSIUM SORBATE, CITRIC ACID, PHENOXYETHANOL, SODIUM BENZOATE, SORBIC ACID.	
Clinique Smart Night™ Custom-Repair Moisturizer	WATER/AQUA/EAU, ISOSTEARYL NEOPENTANOATE, GLYCERIN, BUTYLENE GLYCOL, GLYCERYL STEARATE, PEG-100 STEARATE, DIMETHICONE, CETEARYL ALCOHOL, TAMARINDUS INDICA SEED EXTRACT, BIOTIN, **ERGOTHIONEINE**, CENTAURIUM ERYTHRAEA (CENTAURY) EXTRACT, SIGESBECKIA ORIENTALIS (ST. PAUL'S WORT) EXTRACT, CAFFEINE, WHEY PROTEINLACTIS PROTEINPROTÉINE DU PETIT-LAIT, GLYCINE SOJA (SOYBEAN) PROTEIN, ISOHEXADECANE, HYDROLYZED SOY PROTEIN, ALGAE EXTRACT, SACCHAROMYCES FERMENT FILTRATE, ACETYL HEXAPEPTIDE-8, MICROCOCCUS LYSATE, LECITHIN, YEAST EXTRACTFAEXEXTRAIT DE LEVURE, SOY AMINO ACIDS, CAMELLIA SINENSIS LEAF EXTRACT, ASTROCARYUM MURUMURU SEED BUTTER, GLYCERETH-26, CETYL ALCOHOL, TOCOPHERYL ACETATE, ACETYL GLUCOSAMINE, PETROLATUM,	https://www.clinique.com.pl/

	ACRYLAMIDE/SODIUM ACRYLOYLDIMETHYLTAURATE COPOLYMER, CETEARYL GLUCOSIDE, CAPRYLYL GLYCOL, SODIUM HYALURONATE, ACRYLATES/C10-30 ALKYL ACRYLATE CROSSPOLYMER, SODIUM BENZOATE, TROMETHAMINE, CITRIC ACID, POLYSORBATE 80, AMINOPROPYL ASCORBYL PHOSPHATE, HEXYLENE GLYCOL, HYDROXYETHYLCELLULOSE, SODIUM CITRATE, DISODIUM EDTA, PHENOXYETHANOL	
DR. JETSKE ULTEE Moisturizer normal to dry skin	AQUA, CYCLOPENTASILOXANE, ASCORBYL, TETRAISOPALMITATE, NIACINAMIDE, ETHYLHEXYL, STEARATE, GLYCERIN, CETYL ALCOHOL, GLYCERYL, STEARATE, HYDROGENATED POLYDECENE, SODIUM PCA, CETEARYL ALCOHOL, PEG-75 STEARATE, CAPRYLYL, GLYCOL, TOCOPHEROL, SODIUM, HYALURONATE, SODIUM CARBOXYMETHYL, BETAGLUCAN, **ERGOTHIONEINE**, CETETH-20, STEARETH-20, P-ANISIC ACID, HYDROXYETHYL ACRYLATE/SODIUM ACRYLOYLDIMETHYL TAURATE COPOLYMER, SQUALANE, XANTHAN GUM, SORBITAN ISOSTEARATE, POLYSORBATE 60	https://www.dr-jetskeultee.com/
REVOLVE High Performance Face Cream	WATER, SQUALANE, GLYCERIN, HYDROGENATED ETHYLHEXYL OLIVEATE, HYDROGENATED UNSAVOIDABLE OLIVE OIL, CETYL ALCOHOL, TETRAHEXYLDECYL ASCORBATE, UNSAVOIDABLE HELIANTHUS ANNUUS (SUNFLOWER) SEED OIL, ACETYLGLUCOSAMINE, STEARIC ACID, SORBITAN STEARATE, COPPER LYSINATE, RESUVERATROL, SCENEDESMUS RUBESCENS MICROALGAE EXTRACT, PLANKTON EXTRACT, ARABIDOPSIS THALIANA EXTRACT, MICROCOCCUS EXTRACT, FLOWER/LEAF EXTRACT LEONTOPODIUM ALPINUM (EDELWEISS), **ERGOTHIONEINE**, COFFEA ARABICA (COFFEE) SEED EXTRACT, ILEX PARAGUARIENSIS (YERBA MATE) LEAF EXTRACT, CHRYSANTHEMUM PARTHENIUM (FEVERFEW) EXTRACT, THEOBROMA CACAO (COCOA) EXTRACT, PALMARIA PALMATA RED ALGAE EXTRACT, TREMELLA FUCIFORMIS MUSHROOM EXTRACT, CAFFEINE, BISABOLOL, SODIUM LACTATE, ARGININE, ASPARGINIC ACID, GLYCINE, ALANINE, SERINE, VALINE, PROLINE, THREONINE, ISOLEUCINE, HISTIDINE, PHENYLALANINE, PCA, SODIUM PCA, CHOLESTEROL, PHOSPHATIDYLCHOLINE, LECITHIN, METHYLGLUCOSIDE PHOSPHATE, CAPRYLIC HYDROXAMIC ACID, GLYCERYL CAPRYLATE, ALCOHOL, XANTHAM GUM, CITRIC ACID, SODIUM STEARYL GLUTAMATE, SODIUM METHYLSTEARYL TAURATE, PHENOXYETHANOL, HYDROXYPROPYLMETHYLCELLULOSE STEAROXY ETHER, SODIUM PHYTATE, ETHYLHEXYLGLYCERIN, TOCOPHERYL ACETATE	https://www.revolve.com/
Epionce MelanoLyte Tx	WATER, BUTYLENE GLYCOL, TETRAHEXYLDECYL ASCORBATE, LIMNANTHES ALBA (MEADOWFOAM)	https://www.epionce.pl/

Brightening cream	SEED OIL, UNDECYLENOYL PHENYLALANINE, AMMONIUM ACRYLOYLDIMETHYLTAURATE/VP COPOLYMER, DIPROPYLENE GLYCOL, BEHENYL ALCOHOL, STEARYL ALCOHOL, ASCOPHYLLUM NODOSUM EXTRACT, BISABOLOL, C12-15 ALKYL BENZOATE, CAPRYLIC/CAPRIC TRIGLYCERIDE, CAPRYLYL GLYCOL, CETYL ALCOHOL, CITRIC ACID, CITRUS SINENSIS PEEL OIL EXPRESSED, CRITHMUM MARITIMUM EXTRACT, DISODIUM EDTA, **ERGOTHIONEINE**, ETHYLHEXYLGLYCERIN, GLYCERYL STEARATE, GLYCYRRHIZA GLABRA (LICORICE) ROOT EXTRACT, HEXYLENE GLYCOL, HYDROGENATED LECITHIN, HYDROGENATED VEGETABLE OIL, LINUM USITATISSIMUM (LINSEED) SEED OIL, NYMPHAEA ALBA FLOWER EXTRACT, OLEA EUROPAEA (OLIVE) FRUIT OIL, PEG-20 SOY STEROL, PERSEA GRATISSIMA (AVOCADO) OIL, PHENOXYETHANOL, PHOENIX DACTYLIFERA (DATE) FRUIT EXTRACT, PHYTOSTEROLS, PYRUS MALUS (APPLE) FRUIT EXTRACT, ROSA CANINA FRUIT OIL, SALIX ALBA (WILLOW) BARK EXTRACT, SODIUM HYDROXIDE, SODIUM PALMITOYL PROLINE.	
BasicLab Esteticus Antioxidant Strengthening Serum 0.5% Ergothioneine, 5% Gallic Acid Diglucoside Complex, EGCG - Radiance and Elasticity Day/Night	AQUA, CAPRYLIC/CAPRIC SUCCINIC TRIGLYCERIDE, PENTYLENE GLYCOL, PROPANEDIOL, GLYCERIN, UBIQUINONE, **ERGOTHIONEINE**, DIGLUCOSYL GALLIC ACID, EPIGALLOCATECHIN GALLATE, SH-DECAPEPTIDE-5 SP, GLUCONOLACTONE, MALTODEXTRIN, MANNITOL, CELLULOSE, CELLULOSE GUM, XANTHAN GUM, BENZOIC ACID, SODIUM BENZOATE, HYDROXYACETOPHENONE.	https://basiclab.shop/
BasicLab Esteticus Antioxidant Strengthening Serum 1% Ergothioneine, 10% Complex	AQUA, GLYCERIN, CAPRYLIC/CAPRIC SUCCINIC TRIGLYCERIDE, PENTYLENE GLYCOL, PROPANEDIOL, **ERGOTHIONEINE**, UBIQUINONE, DIGLUCOSYL GALLIC ACID, EPIGALLOCATECHIN GALLATE, EPILOBIUM FLEISCHERI LEAF/STEM EXTRACT, SH-DECAPEPTIDE-5 SP, GLUCONOLACTONE, ARGININE, MALTODEXTRIN, MANNITOL, CELLULOSE, CELLULOSE GUM, XANTHAN GUM, CITRIC ACID, BENZOIC ACID, SODIUM BENZOATE, HYDROXYACETOPHENONE.	https://basiclab.shop/
Photozyme Dna Youth Recovery Facial Serum	WATER, ETHYLHEXYL ISONONANOATE, GLYCERIN, ISONONYL ISONONANOATE, PROPANEDIOL, HORDEUM DISTICHON (BARLEY) EXTRACT, **ERGOTHIONEINE**, SODIUM HYALURONATE, PHELLODENDRON AMURENSE BARK EXTRACT, SANTALUM, ALBUM (SANDALWOOD) EXTRACT, PLANKTON EXTRACT, ARABIDOPSIS THALIANA EXTRACT, EVODIA RUTAECAPRA FRUIT EXTRACT, LECITHIN, MICROCOCCUS LYSATE, BUTYLENE GLYCOL, ACRYLATES/C10-30 ALKYL ACRYLATE, CROSSPOLYMER, CARBOMER, BHT, DISODIUM EDTA, PHENOXYETHANOL, ETHYLHEXYLGLYCERIN, SODIUM HYDROXIDE	https://photozyme.com/
DoSe By VH Azelaic Acid 10% Serum	AQUA (WATER), AZELAIC ACID, PROPANEDIOL, LACTOBACILLUS FERMENT, GLYCERIN, SILICA,	https://victoriahealth.com/

	**ERGOTHIONEINE**, SODIUM HYALURONATE, ALLANTOIN, SODIUM GLUCONATE, SODIUM LACTATE, CARBOMER, CAPRYLYL GLYCOL, HYDROLYZED JOJOBA PROTEIN, HYDROGENATED PHOSPHATIDYLCHOLINE, DEHYDROXANTHAN GUM, POTASSIUM SORBATE, LEUCONOSTOC/RADISH ROOT FERMENT FILTRATE, SODIUM HYDROXIDE, PHENOXYETHANOL	
NuFACE<br>NuFACE Protect and Tighten Super Antioxidant Booster Serum	WATER, PROPANEDIOL, GLYCERIN, 1,2-HEXANEDIOL, PENTYLENE GLYCOL, BUTYLENE GLYCOL, ARTEMISIA CAPILLARIS FLOWER EXTRACT, CAMELLIA SINENSIS LEAF EXTRACT, CARNOSINE, CARRAGEENAN, CITRIC ACID, COPPER GLUCONATE, DIMETHYLMETHOXY CHROMANOL, **ERGOTHIONEINE**, HYDROXYACETOPHENONE, HYDROXYACETOPHENONE, LACTOBACILLUS FERMENT, LEONTOPODIUM ALPINUM CALLUS CULTURE EXTRACT, LYCIUM CHINENSE FRUIT EXTRACT, MAGNESIUM ASPARTATE, MALTODEXTRIN, PULLULAN, SCLEROTIUM GUM, SEA SILT EXTRACT, TRISODIUM ETHYLENEDIAMINE DISUCCINATE, VACCINIUM ANGUSTIFOLIUM (BLUEBERRY) FRUIT EXTRACT, VACCINIUM MACROCARPON (CRANBERRY) FRUIT EXTRACT, XANTHAN GUM, YEAST EXTRACT, ZINC GLUCONATE.	https://www.mynuface.com/
Paula’s Choice 25% Vitamin C + Glutathione Clinical Serum	AQUA/WATER/EAU, TETRAHEXYLDECYL ASCORBATE, DIMETHICONE, GLYCERIN, C9-12 ALKANE, ASCORBYL GLUCOSIDE, SILICA, PYRUS MALUS (APPLE) FRUIT EXTRACT, HYDROGENATED LECITHIN, POLYGLYCERYL-6 POLYRICINOLEATE, ACRYLATES/C10-30 ALKYL ACRYLATE CROSSPOLYMER, XANTHAN GUM, ORYZA SATIVA (RICE) BRAN EXTRACT, HYDROXYETHYL ACRYLATE/SODIUM ACRYLOYLDIMETHYL TAURATE COPOLYMER, CETYL ALCOHOL, CAPRYLYL GLYCOL, LYSINE CARBOXYMETHYL CYSTEINATE, COCO CAPRYLATE/CAPRATE, TOCOPHEROL, GLUTATHIONE, SODIUM CITRATE, SODIUM PHYTATE, HEXYLENE GLYCOL, ETHYLHEXYLGLYCERIN, HELIANTHUS ANNUUS SUNFLOWER SEED EXTRACT, UREA, MANNITOL, CAPRYLIC/CAPRIC TRIGLYCERIDE, SODIUM PCA, ROSMARINUS OFFICINALIS (ROSEMARY) LEAF EXTRACT, POLYSORBATE 60, SORBITAN ISOSTEARATE, TREHALOSE, ASCORBIC ACID, GLUCOSE, VANILLA PLANIFOLIA FRUIT EXTRACT, POLYQUATERNIUM-51, **ERGOTHIONEINE**, TRIACETIN, PHENOXYETHANOL.	https://www.paulaschoice.pl/
DoSe By VH<br>Vitamin C Eye Cream	AQUA (WATER), PROPANEDIOL, GLYCERIN, RICINUS COMMUNIS (CASTOR) SEED OIL, TETRAHEXYLDECYL ASCORBATE, PRUNUS AMYGDALUS DULCIS (SWEET ALMOND) OIL, C14-22 ALCOHOLS, PENTYLENE GLYCOL, BUTYROSPERMUM PARKII (SHEA) BUTTER, HYDROXYETHYL ACRYLATE/SODIUM ACRYLOYLDIMETHYL TAURATE COPOLYMER, CAFFEINE, PALMITOYL TRIPEPTIDE-1, PALMITOYL	https://victoriahealth.com/

	TRIPEPTIDE-5, PALMITOYL TETRAPEPTIDE-7, GLYCINE SOJA (SOYBEAN) PROTEIN, SODIUM HYALURONATE, HYDROLYZED RICE PROTEIN, ERGOTHIONEINE, SORBITAN ISOSTEARATE, NYMPHAEA CAERULEA (BLUE LOTUS) FLOWER EXTRACT, LONICERA JAPONICA (HONEYSUCKLE) FLOWER EXTRACT, LONICERA CAPRIFOLIUM (HONEYSUCKLE) FLOWER EXTRACT, CARBOMER, CAPRYLYL GLYCOL, GLUCOSE, C12-20 ALKYL GLUCOSIDE, COCO-CAPRYLATE/CAPRATE, XANTHAN GUM, POLYSORBATE 20, BUTYLENE GLYCOL, POLYSORBATE 60, SUPEROXIDE DISMUTASE, POTASSIUM SORBATE, SODIUM DEXTRAN SULFATE, PHENOXYETHANOL, SODIUM BENZOATE, SODIUM HYDROXIDE, CITRIC ACID.	
Sesderma Reti Age Eye Contour Gel Eye Cream with Retinol	AQUA, CAPRYLIC/CAPRIC TRIGLYCERIDE, ISOSTEARYL ISOSTEARATE, PROPANEDIOL, PENTYLENE GLYCOL, PEG-100 STEARATE, GLYCERYL STEARATE, PROPYLENE GLYCOL, GLYCERIN, NICOTIANA BENTHAMIANA HEXAPEPTIDE-40 SH-POLYPEPTIDE-76, NICOTIANA BENTHAMIANA SH-POLYPEPTIDE-15 HEXAPEPTIDE-40, AESCULUS HIPPOCASTANUM BARK EXTRACT, ALCOHOL, ALPHA-ARBUTIN, ASCORBYL GLUCOSIDE, BHA, BHT, BUTYLENE GLYCOL, CAMPHANEDIOL, CAPROOYL TETRAPEPTIDE-3, CARBOMER, CETEARYL ALCOHOL, CI 17200, DEXTRAN, DIPOTASSIUM PHOSPHATE, DISODIUM EDTA**, ERGOTHIONEINE**, ETHYLHEXYLGLYCERIN, GINKGO BILOBA LEAF EXTRACT, GLYCINE SOJA PROTEIN, HELIANTHUS ANNUUS SEED OIL, HYDROCHLORIC ACID, HYDROLYZED RICE BRAN PROTEIN, HYDROLYZED SODIUM HYALURONATE, LECITHIN, PARFUM, PHENOXYETHANOL, PINANEDIOL, POLYSORBATE 20, POTASSIUM PHOSPHATE, POTASSIUM SORBATE, RESVERATROL DIMETHYL ETHER, RETINAL, RETINOL, RETINYL PROPIONATE, SODIUM BENZOATE, SODIUM CHLORIDE, SODIUM CHOLATE, SODIUM DEXTRAN SULFATE, SODIUM HYDROXIDE, SUPEROXIDE DISMUTASE, TRIETHANOLAMINE, TROMETHAMINE, ZINC CHLORIDE.	https://www.sesderma.com/pl
Dermaquest Peptide 3D Eye Lift	WATER, CAPRYLIC/CAPRIC TRIGLYCERIDE, GLYCERIN, CETEARYL OLIVATE, SORBITAN OLIVATE, GLYCERYL STEARATE, PEG-100 STEARATE, CETYL ALCOHOL, PPG-12/SMDI COPOLYMER, SODIUM ASCORBYL PHOSPHATE, ALEURITES MOLUCCANA SEED OIL, DISODIUM EDTA, ALLANTOIN, BUTYROSPERMUM PARKII NUT EXTRACT, TOCOPHERYL ACETATE, ASCORBYL PALMITATE, TOCOPHEROL, XANTHAN GUM, MAGNESIUM ALUMINUM SILICATE, CRITHMUM MARITIMUM EXTRACT, PHENOXYETHANOL, PICHIA/RESVERATROL FERMENT EXTRACT, ERYNGIUM MARITIMUM EXTRACT, HYDROXYPROPYL CYCLODEXTRIN, PALMITOYL TRIPEPTIDE-38, GARDENIA JASMINOIDES MERISTEM CELL, ARTEMISIA VULGARIS EXTRACT, LAMINARIA	https://dermaquest.pl/

Estée Lauder Re-Nutriv Ultimate Lift Regenerating Youth Eye Crème	DIGITATA EXTRACT, PORPHYRIDIUM POLYSACCHARIDE, TREMELLA FUCIFORMIS SPOROCARP EXTRACT, BIOSACCHARIDE GUM-1, PROPYLENE GLYCOL, HYDROLYZED GLYCOSAMINOGLYCANS, CAPRYLYL GLYCOL, HEXYLENE GLYCOL, SODIUM HYALURONATE, CHONDRUS CRISPUS EXTRACT, CITRIC ACID, POTASSIUM SORBATE, **ERGOTHIONEINE**, ACETYL HEXAPEPTIDE-8, SODIUM CARBOXYMETHYL BETAGLUCAN, IMIDAZOLIDINYL UREA, ETHYLHEXYLGLYCERIN, METHYLGLUCOSIDE PHOSPHATE, COPPER LYSINATE/PROLINATE, BUTYLENE GLYCOL, HYDROLYZED VEGETABLE ADENOSINE TRIPHOSPHATE, SODIUM CHONDROITIN SULFATE, VANILLA PLANIFOLIA FRUIT EXTRACT, SODIUM HYDROXIDE.<br>WATER AQUA EAU, CYCLOPENTASILOXANE, BUTYLENE GLYCOL, GLYCERIN, DIMETHICONE, CAPRYLIC/CAPRIC/MYRISTIC/STEARIC TRIGLYCERIDE, POLYSILICONE-11, SWERTIA CHIRATA EXTRACT, NARCISSUS TAZETTA BULB EXTRACT, LAMINARIA DIGITATA EXTRACT, SODIUM HYALURONATE, ANTHEMIS NOBILIS (CHAMOMILE) FLOWER EXTRACT, CORDYCEPS SINENSIS EXTRACT, FUSCOPORIA OBLIQUA EXTRACT, CUCUMIS SATIVUS (CUCUMBER) FRUIT EXTRACT, SACCHAROMYCES LYSATE EXTRACT, HYPNEA MUSCIFORMIS (ALGAE) EXTRACT, ASPARAGOPSIS ARMATA EXTRACT, ALGAE EXTRACT, PORPHYRIDIUM CRUENTUM EXTRACT, ASCOPHYLLUM NODOSUM EXTRACT, LAMINARIA OCHROLEUCA EXTRACT, SCUTELLARIA BAICALENSIS ROOT EXTRACT, TOCOPHERYL ACETATE, EUPHRASIA OFFICINALIS (EUPHRASIA) EXTRACT, ACETYL HEXAPEPTIDE-8, GELIDIELLA ACEROSA EXTRACT, HELIANTHUS ANNUUS (SUNFLOWER) SEED EXTRACT, ARTEMIA EXTRACT, PYRUS MALUS (APPLE) FRUIT EXTRACT, HORDEUM VULGARE (BARLEY) EXTRACT EXTRAIT D'ORGE, CETYL PEG/PPG-10/1 DIMETHICONE, CYCLOHEXASILOXANE, HYDROGENATED POLYISOBUTENE, ISONONYL ISONONANOATE, TREHALOSE, SORBITOL, ACETYL GLUCOSAMINE, CAPRYLYL GLYCOL, CAFFEINE, LAURYL PCA, CAPRYLIC/CAPRIC TRIGLYCERIDE, LAURYL PEG-9 POLYDIMETHYLSILOXYETHYL DIMETHICONE, WHEY PROTEIN LACTIS PROTEIN PROTEINE DU PETIT-LAIT, POLYGONUM CUSPIDATUM ROOT EXTRACT, GOLD, HYDROLYZED WHEAT PROTEIN, DECARBOXY CARNOSINE HCL, PROPYLENE GLYCOL DICAPRATE, PEARL POWDER, **ERGOTHIONEINE**, LINOLEIC ACID, GLUCOSE, GLYCINE SOJA (SOYBEAN) PROTEIN, YEAST EXTRACT FAEX EXTRAIT DE LEVURE, SUCROSE, CITRIC ACID, HEXYLENE GLYCOL, TRIETHOXYCAPRYLYLSILANE,	https://www.esteelauder.pl/

	POLYMETHYLSILSESQUIOXANE, CELLULOSE, PEG/PPG-18/18 DIMETHICONE, ALUMINA, NYLON-12, CYCLODEXTRIN, SILICA, DIMETHICONE CROSSPOLYMER, LECITHIN, MAGNESIUM SULFATE, BHT, ETHYLBISIMINOMETHYLGUAIACOL MANGANESE CHLORIDE, SODIUM SULFITE, SODIUM METABISULFITE, DISODIUM EDTA, PHENOXYETHANOL, SODIUM BENZOATE, POTASSIUM SORBATE, MICA, TITANIUM DIOXIDE (CI 77891), IRON OXIDES (CI 77491) <ILN47948>	
grace & stella Brightening eye stick	AQUA/WATER/EAU, PROPYLENE GLYCOL, GLYCERIN, ROSA RUGOSA (ROSE) FLOWER WATER, ISOPROPYL MYRISTATE, SQUALANE, CAFFEINE, NIACINAMIDE, CETYL ALCOHOL, CETYL ETHYLHEXANOATE, SYNTHETIC BEESWAX, CAPRYLIC/CAPRIC TRIGLYCERIDE, DIMETHICONE, BUTYLENE GLYCOL, HYDROGENATED LECITHIN, SODIUM LAUROYL LACTYLATE, TOCOPHEROL, BUTYROSPERMUM PARKII (SHEA) BUTTER, 1,2-HEXANEDIOL, TRIETHYL CITRATE, CARBOMER, POLYSORBATE 20, POLYACRYLATE CROSSPOLYMER-6, CERAMIDE NP, ROSA RUGOSA (ROSE) FLOWER OIL, SODIUM LACTATE, PANTHENOL, XANTHAN GUM, PALMITOYL TRIPEPTIDE-1, PALMITOYL TETRAPEPTIDE-7, SODIUM HYALURONATE, **ERGOTHIONEINE**, HYDROXYPROPYL TETRAHYDROPYRANTRIOL, BISABOLOL, CROCUS SATIVUS FLOWER EXTRACT	https://www.graceandstella.com/
Allies of Skin Molecular Saviour Probiotics Treatment Mist	AQUA (WATER), POLYSORBATE 20, NIACINAMIDE, PROPANEDIOL, LACTOBACILLUS FERMENT, GLUCONOLACTONE, MANGIFERA INDICA FRUIT EXTRACT, LACTOBACILLUS FERMENT LYSATE FILTRATE, YOGURT, ZINC PCA, BISABOLOL, EUTERPE OLERACEA FRUIT EXTRACT, CUCUMIS SATIVUS FRUIT EXTRACT, ALOE BARBADENSIS LEAF JUICE POWDER, COCODIMONIUM HYDROXYPROPYL SILK AMINO ACIDS, SILVER, CITRUS AURANTIUM DULCIS CALLUS CULTURE EXTRACT, **ERGOTHIONEINE**, GLUTATHIONE, GLYCERIN, POPULUS TREMULOIDES BARK EXTRACT, LEUCONOSTOC/RADISH ROOT FERMENT FILTRATE, PALMITOYL TRIPEPTIDE-1, PALMITOYL TETRAPEPTIDE-7, CHRYSIN, N-HYDROXYSUCCINIMIDE, GLUTAMYLAMIDOETHYL IMIDAZOLE, XANTHAN GUM, CALCIUM GLUCONATE, ETHYLHEXYLGLYCERIN, SORBIC ACID, SODIUM BENZOATE, SODIUM GLUCONATE, STEARETH-20, SODIUM HYDROXIDE, PHENOXYETHANOL.	https://eu.allies.shop/
SESDERMA REPASKIN DEFENSE Mist	AQUA, GLYCERIN, PROPYLENE GLYCOL, PROPANEDIOL, BIS-PEG-18 METHYLETHER DIMETHYL SILANE, LECITHIN, PEG-40 HYDROGENATED CASTOR OIL, ALCOHOL, CAMELLIA SINENSIS LEAF EXTRACT, SYLIBUM MARIANUM EXTRACT, RESVERATROL DIMETHYL ETHER, VITIS VINIFERA VINE EXTRACT, THIOCTIC AC., QUERCETIN, GENISTEIN GLUCOSIDE, GLUTATHIONE, GLYCINE SOJA SEED EXTRACT,	https://www.sesderma.com/

	UBIQUINONE, SUPEROXIDE DISMUTASE, **ERGOTHIONEINE**, CHONDRUS CRISPUS EXTRACT, POLYSORBATE 20, BUTYLENE GLYCOL, TOCOPHERYL ACETATE, HYDROLYZED JOJOBA ESTERS, SODIUM CHOLATE, DISODIUM EDTA, BHT, SODIUM CHLORIDE, INULIN, PENTYLENE GLYCOL, GLYCINE SOJA OIL, PHOSPHOLIPIDS, CPARYLYL GLYCOL, XANTAN GUM, TROMETHAMINE, SODIUM BISULFITE, CITRIC AC., PHENOXYETHANOL, ETHYLHEXYLGLYCERIN, POTASSIUM SORBATE, PARFUM, CITRONELLYL METHYLCROTONATE.	
AMPLEUR Luxury White W Protect UV+ SPF50 Pa++++	WATER, ETHYLHEXYL METHOXYCINNAMATE, BUTYLENE GLYCOL, CYCLOPENTASILOXANE, METHYL METHACRYLATE CROSSPOLYMER, ALCOHOL, BUTYL METHOXYDIBENZOYLMETHANE, POLYSILICONE-14, ISOTRIDECYL ISONONANOATE, LAURETH-9, PEG-8 GLYCERYL ISOSTEARATE, HYDROXYETHYL ACRYLATE/SODIUM ACRYLOYLDIMETHYL TAURATE COPOLYMER, ACRYLATES/C10-30 ALKYL ACRYLATE CROSSPOLYMER, SQUALANE, AMINOMETHYL PROPANOL, FRAGRANCE, POLYSORBATE 60, TOCOPHEROL, STEARYL GLYCYRRHETINATE, MANNITOL, PLACENTAL PROTEIN, GLYCERIN, POLYQUATERNIUM-51, GENTIANA LUTEA ROOT EXTRACT, ARGININE, OENOTHERA BIENNIS (EVENING PRIMROSE) SEED EXTRACT, PHENOXYETHANOL, MORINGA PTERYGOSPERMA SEED EXTRACT, LECITHIN, PUERARIA THUNBERGIANA ROOT EXTRACT, PLANKTON EXTRACT, HYDROLYZED COLLAGEN, ALOE BARBADENSIS LEAF EXTRACT, CERAMIDE NG, CHLORELLA VULGARIS EXTRACT, HYDROGENATED LECITHIN, ERGOTHIONEINE, CARTHAMUS TINCTORIUS (SAFFLOWER) SEED OIL, GLYCINE SOJA (SOYBEAN) STEROLS, XANTHOPHYLL	https://ampleur.id/
Nevoa Active Optimal Defense I Broad Spectrum SPF 43	ACTIVE INGREDIENTS: OCTINOXATE 7.5%, ZINC OXIDE 9.0%. INACTIVE INGREDIENTS: ASCORBYL PALMITATE, BUTYLENE GLYCOL, CITRIC ACID, CYCLOPENTASILOXANE, DIMETHICONE, DIMETHICONE/PEG-10/15 CROSSPOLYMER, DIMETHICONE/VINYLDIMETHICONE CROSSPOLYMER, **ERGOTHIONEINE**, ETHYL HEXYL ISONONANOATE, IODOPROPYNYL BUTYLCARBAMATE, LAURYL PEG-9 POLYMETHYLSILOXYETHYL DIMETHICONE, LECITHIN, MICROCOCCUS LYSATE, PHENOXYETHANOL, PLANKTON EXTRACT, PURIFIED WATER, RETINYL PALMITATE, SODIUM CHLORIDE, SODIUM HYDROXIDE, TRIETHOXYCAPRYLYLSILANE.	https://www.neova.com/
Prep + Prime Face Protect Lotion SPF 50	WATER AQUA EAU, HOMOSALATE, ETHYLHEXYL SALICYLATE, BUTYLOCTYL SALICYLATE, METHYL TRIMETHICONE, NEOPENTYL GLYCOL DIHEPTANOATE, BUTYL METHOXYDIBENZOYLMETHANE, BENZOPHENONE-3 (OXYBENZONE), ALEURITES	https://www.maccosmetics.pl/

	MOLUCCANA (KUKUI) SEED OIL, OCTOCRYLENE, LAURYL PEG-9 POLYDIMETHYLSILOXYETHYL DIMETHICONE, PEG-100 STEARATE, BUTYLENE GLYCOL, GLYCERYL STEARATE, DIPENTAERYTHRITYL TRI-POLYHYDROXYSTEARATE, SODIUM HYALURONATE, LAVANDULA ANGUSTIFOLIA (LAVENDER) FLOWER WATER, LAMINARIA OCHROLEUCA EXTRACT, LAMINARIA DIGITATA EXTRACT, ALGAE EXTRACT, CAFFEINE, ETHYLHEXYLGLYCERIN, CAPRYLIC/CAPRIC TRIGLYCERIDE, SORBITOL, METHYLDIHYDROJASMONATE, **ERGOTHIONEINE**, DEHYDROXANTHAN GUM, VP/EICOSENE COPOLYMER, SACCHARIDE ISOMERATE, AMMONIUM ACRYLOYLDIMETHYLTAURATE/VP COPOLYMER, POTASSIUM CETYL PHOSPHATE, CAPRYLYL GLYCOL, CITRIC ACID, STEARIC ACID, CETYL ALCOHOL, LINALOOL, LIMONENE, DISODIUM EDTA, PHENOXYETHANOL, SODIUM DEHYDROACETATE, MICA <ILN40728>	
Dr Irena Eris BB CREAM SPF 50 Waterproof Tinted Moisturizer	AQUA (WATER), DIMETHICONE, TITANIUM DIOXIDE (NANO), ETHYLHEXYL METHOXYCINNAMATE, DICAPRYLYL CARBONATE, ISODODECANE, CI 77891 (TITANIUM DIOXIDE), ALCOHOL DENAT., CI 77492 (IRON OXIDES), BUTYLENE GLYCOL, NIACINAMIDE, TRIMETHYLSILOXYSILICATE, VINYL DIMETHICONE/METHICONE SILSESQUIOXANE CROSSPOLYMER, LAURYL PEG-9 POLYDIMETHYLSILOXYETHYL DIMETHICONE, METHYL TRIMETHICONE, ACRYLATES/ DIMETHICONE COPOLYMER, CITRUS AURANTIUM DULCIS (ORANGE) FRUIT EXTRACT, **ERGOTHIONEINE**, TOCOPHEROL, ALUMINUM HYDROXIDE, STEARIC ACID, DISTEARDIMONIUM HECTORITE, DIMETHICONE/PEG-10/15 CROSSPOLYMER, SODIUM CHLORIDE, PROPYLENE CARBONATE, SODIUM CITRATE, PERFLUOROOCTYL TRIETHOXYSILANE, ETHYLHEXYLGLYCERIN, POLYPERFLUOROMETHYLISOPROPYL ETHER, PHENOXYETHANOL, DIPROPYLENE GLYCOL, PARFUM (FRAGRANCE), HEXAMETHYLINDANOPYRAN, LINALOOL, LINALYL ACETATE, ACETYL CEDRENE, LIMONENE, CITRUS AURANTIUM BERGAMIA PEEL OIL, CITRUS AURANTIUM PEEL OIL, POGOSTEMON CABLIN OIL, BENZYL SALICYLATE, HEXYL CINNAMAL, ALPHA-ISOMETHYL IONONE, CITRONELLOL, GERANYL ACETATE, PINENE, CI 77491 (IRON OXIDES), CI 77499 (IRON OXIDES)	https://www.drirenaeris.com/
Estée Lauder Perfectionist Pro Multi-Defense Aqua UV Gel SPF 50 with 8 Anti-Oxidants	AVOBENZONE 3.0%, HOMOSALATE 5.0%, OCTISALATE 4.5%, OCTOCRYLENE 2.8%, OXYBENZONE 3.0%INGREDIENTS: WATER\AQUA\EAU, ALCOHOL DENAT., BUTYLENE GLYCOL, TOCOPHERYL ACETATE, SODIUM HYALURONATE, CAMELLIA SINENSIS (GREEN TEA) LEAF EXTRACT, ANTHEMIS NOBILIS (CHAMOMILE) FLOWER EXTRACT, ALGAE EXTRACT,	https://www.esteelauder.pl/

	TRITICUM VULGARE (WHEAT) GERM EXTRACT, LAMINARIA OCHROLEUCA EXTRACT, POLYGONUM CUSPIDATUM ROOT EXTRACT, SQUALANE, HORDEUM VULGARE (BARLEY) EXTRACT\ EXTRAIT D'ORGE, LILIUM TIGRINUM EXTRACT, OPUNTIA TUNA EXTRACT, CAFFEINE, ACRYLATES COPOLYMER, CAPRYLIC/CAPRIC TRIGLYCERIDE, CAPRYLYL GLYCOL, CHOLESTEROL, **ERGOTHIONEINE**, GLYCERIN, SUCROSE, ALCALIGENES POLYSACCHARIDES, THERMUS THERMOPHILLUS FERMENT, SORBITAN SESQUIISOSTEARATE, TETRAHEXYLDECYL ASCORBATE, HEXYLENE GLYCOL, ACRYLATES/BEHENETH-25 METHACRYLATE COPOLYMER, SODIUM HYDROXIDE, DEHYDROXANTHAN GUM, DIPROPYLENE GLYCOL, CARBOMER, PPG-8-CETETH-20, DEXTRIN PALMITATE, CYCLODEXTRIN, ACRYLATES/C10-30 ALKYL ACRYLATE CROSSPOLYMER, SORBETH-30 TETRAISOSTEARATE, GLYCERYL STEARATE, POLYMETHYLSILSESQUIOXANE, ACRYLOYLDIMETHYLTAURATE/VP COPOLYMER, BHT, ETHYLBISIMINOMETHYLGUAIACOL MANGANESE CHLORIDE, DISODIUM EDTA, PHENOXYETHANOL <ILN47518>	
SHISEIDO Urban Environment Mineral Clear Sunscreen SPF 50	ACTIVE INGREDIENTS: TITANIUM DIOXIDE 4.5%, ZINC OXIDE 17.2% INACTIVE INGREDIENTS: DIMETHICONE, WATER, DIISOPROPYL SEBACATE, DIPHENYLSILOXY PHENYL TRIMETHICONE, DIPROPYLENE GLYCOL, GLYCERIN, SORBITAN SESQUIISOSTEARATE, POLYHYDROXYSTEARIC ACID, LAURYL PEG-9 POLYDIMETHYLSILOXYETHYL DIMETHICONE, SILICA, AMINOETHANESULFINIC ACID, THIOTAURINE, SCUTELLARIA BAICALENSIS ROOT EXTRACT, PAEONIA ALBIFLORA ROOT EXTRACT, SODIUM HYALURONATE, DIPOTASSIUM GLYCYRRHIZATE, GINKGO BILOBA LEAF EXTRAC, PEG/PPG-14/7 DIMETHYL ETHER, ONONIS SPINOSA ROOT EXTRACT, CAMELLIA JAPONICA FLOWER EXTRACT, GARCINIA MANGOSTANA BARK EXTRACT, TREHALOSE, SPIRULINA PLATENSIS EXTRACT, SOLUBLE COLLAGEN, ALUMINUM HYDROXIDE, TRIETHOXYCAPRYLYLSILANE, STEARIC ACID, DISTEARDIMONIUM HECTORITE, BUTYLENE GLYCOL, TRISODIUM EDTA, ISOSTEARIC ACID, SODIUM CITRATE, CITRIC ACID, SODIUM METABISULFITE, PRUNUS YEDOENSIS LEAF EXTRACT, SYZYGIUM JAMBOS LEAF EXTRACT, TOCOPHEROL, MICA, DISODIUM PHOSPHATE, RIBOFLAVIN, **ERGOTHIONEINE**, SODIUM PHOSPHATE, PHENOXYETHANOL, BENZOIC ACID, TITANIUM DIOXIDE	https://www.shiseido.com/
SHIKAI 8 oz Borage Therapy Lotion - Original Formula	PURIFIED WATER (AQUA), DECOLORIZED ALOE VERA GEL (ALOE BARBADENSIS), GLYCERYL STEARATE (VEGETABLE EMULSIFIER), SAFFLOWER SEED OIL	https://shikai.com/

(CARTHAMUS TINCTORIUS), GLYCERIN, JOJOBA SEED OIL (SIMMONDSIA CHINENSIS), BORAGE SEED OIL (BORAGO OFFICINALIS), CETYL ALCOHOL (VEGETABLE WAX), VITAMIN E ACETATE (TOCOPHERYL ACETATE), DIMETHICONE, SHEA BUTTER (BUTYROSPERMUM PARKII FRUIT), SODIUM ASCORBYL PHOSPHATE (VITAMIN C), PHENOXYETHANOL, **L-ERGOTHIONEINE** (AMINO ACID), ETHYLHEXYL GLYCERIN.
